# Recent Advances in Chitosan-Based Applications—A Review

**DOI:** 10.3390/ma16052073

**Published:** 2023-03-03

**Authors:** Charitha Thambiliyagodage, Madara Jayanetti, Amavin Mendis, Geethma Ekanayake, Heshan Liyanaarachchi, Saravanamuthu Vigneswaran

**Affiliations:** 1 Faculty of Humanities and Sciences, Sri Lanka Institute of Information Technology, Malabe 10115, Sri Lanka; 2 Faculty of Engineering and Information Technology, University of Technology Sydney, P.O. Box 123, Broadway, NSW 2007, Australia; 3 Faculty of Sciences & Technology (RealTek), Norwegian University of Life Sciences, P.O. Box 5003, N-1432 Ås, Norway

**Keywords:** chitosan, medicine, cosmetic, food, environmental remediation, agriculture

## Abstract

Chitosan derived from chitin gas gathered much interest as a biopolymer due to its known and possible broad applications. Chitin is a nitrogen-enriched polymer abundantly present in the exoskeletons of arthropods, cell walls of fungi, green algae, and microorganisms, radulae and beaks of molluscs and cephalopods, etc. Chitosan is a promising candidate for a wide variety of applications due to its macromolecular structure and its unique biological and physiological properties, including solubility, biocompatibility, biodegradability, and reactivity. Chitosan and its derivatives have been known to be applicable in medicine, pharmaceuticals, food, cosmetics, agriculture, the textile and paper industries, the energy industry, and industrial sustainability. More specifically, their use in drug delivery, dentistry, ophthalmology, wound dressing, cell encapsulation, bioimaging, tissue engineering, food packaging, gelling and coating, food additives and preservatives, active biopolymeric nanofilms, nutraceuticals, skin and hair care, preventing abiotic stress in flora, increasing water availability in plants, controlled release fertilizers, dye-sensitised solar cells, wastewater and sludge treatment, and metal extraction. The merits and demerits associated with the use of chitosan derivatives in the above applications are elucidated, and finally, the key challenges and future perspectives are discussed in detail.

## 1. Introduction

Polysaccharides are mainly classified into two groups: homopolysaccharides and heteropolysaccharides, where the homopolysaccharides are composed of a single type of monomer while the heteropolysaccharides are originated by two or more monosaccharide units. Cellulose, chitin, amylose, amylopectin, glycogen, and dextran are some of the examples of homopolysaccharides and glycans that are present in bacterial cell walls and are made from a heteropolymer of alternating β (1-4) linked N-acetylglucosamine and N-acetylmuramic acid residues. Chitin is the most abundant natural amino polysaccharide and the second most abundant natural polysaccharide, which is comprised of N-acetyl glucosamine residues linked via β (1-4) glycosidic bonds. The structure of chitin is different from that of cellulose, the most abundant polysaccharide, where an acetylated amino group is present at the C2 position instead of the hydroxyl group. Chitin is present in green algae, the cell walls of fungi, the exoskeletons of crustaceans such as shrimps, crab, and lobster, and in the cuticles of insects and arachnids, providing structural integrity [1]. Chitosan is a linear polysaccharide produced by deacetylation of chitin by the hydrolysis of the acetamide groups by strong alkaline treatment. It is composed of β (1-4) linked 2-amino-2-deoxy-β-D-glucopyranose with 2-acetamino-2-deoxy-β-D-glucopyranose.

**Scheme 1:**
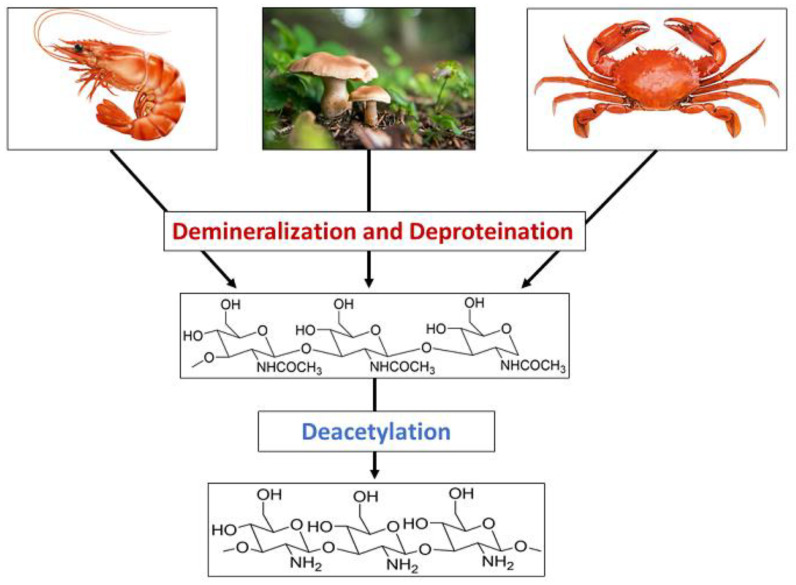
Synthesis of chitosan bio polymer using chitin presenting natural sources.

Chitosan is produced from chitin through a series of chemical reactions, as shown in Scheme 1. The extraction of chitosan mainly involves demineralization, deproteination, and deacetylation. Few studies have reported performing decolorization as a minor step. Chitin could be taken directly from the purified synthetic substance, or it could be taken from natural sources. The most commonly used natural material for such purposes is shrimp shells, and additionally, crab, fungi, etc. have also been used. Shrimp shells are washed, dried, and pulverized to remove any dirt before subjecting them to any chemical treatment. They are comprised of chitin, protein, and minerals such as calcium carbonate and calcium phosphate, which are combined with proteins and chitin and trapped in the exoskeleton [2]. Therefore, treating with organic or inorganic acids is essential to remove the minerals [3,4]. HCl has been used mainly for the demineralization [5,6,7,8], and additionally, inorganic and organic acids such as H_2_SO_4_, HNO_3_, CH_3_COOH, CH_2_O_2_, and HClO_3_ [8,9,10,11] have also been used to remove the calcium salts. During the treatment, pH increases with the release of Ca^2+^ ions, and neutralization after the treatment is essential to stop the demineralization. During the demineralization process, CaCl_2_ and CO_2_ are produced.

Ca_3_(PO_4_)_2_ + 6HCl = 3CaCl_2_ + 2H_3_PO_4_

The acid concentration, extraction temperature, and time are the key factors that determine the efficiency of demineralization and the purity of the chitosan produced. Demineralized chitin is then subjected to deproteination in a diluted alkaline medium. Proteins are harder to remove as they are covalently bound to chitin-forming glycoproteins. Therefore, the deproteination process is comparatively longer and could take more than a day as well. Proteins being linked to chitin limits its applications as proteins trigger the immune response in the immune system, leading to restrictions in using chitin for biological applications. Thus, the removal of proteins is of great importance. Mainly, deproteinated chitin is treated with diluted NaOH, whose concentration varies in the range of 0.5–4 mg/L. In addition to NaOH, other basic chemicals such as KOH, Na_2_CO_3_, Ca(OH)_2_, K_2_CO_3_, and NaHCO_3_ have been used for deproteination [12]. The efficiency of the process depends on the alkaline concentration, temperature, and duration. Huang et al. reported the use of a natural deep eutectic solvent made from choline chloride and malic acid for both the demineralization and deproteination of crab shells. Minerals that are deposited in the chitin-protein matrix of the crab exoskeleton are removed by malic acid, and the strong internal structure is thus weakened. Furthermore, the strong hydrogen bond network between chitin and protein is then less strengthened due to the hydrogen bonds formed between the chloride ions of the solvent and the hydroxyl groups, and hence the proteins are also removed [13]. Deproteination could also be achieved by treating demineralized crab or shrimp shells with proteolytic enzymes, including chymotrypsin, Alkalase, Pepsin, Papain, and Trypsin [14,15,16]. Demineralization should be performed before deproteination to increase tissue permeability, promote the action of the enzymes, and reduce the amount of enzyme inhibitors that may be present [17]. Mhamdi et al. reported the use of the thermostable serine alkaline proteases from *Micromonospora chaiyaphumensis* S103, with which 93% deproteination was achieved with an enzyme/substrate ratio of 20 U/mg [18], and digestive alkaline proteases from the viscera of *Portunus segnis* were used to obtain a deproteination of 85% with crab and 91% with shrimp shells [19]. Lucas et al. reported the deproteination of chitin extracted from the cuticles of insects using Alcalase, which is a bacterial endopeptidase produced from the submerged fermentation of Bacillus licheniformis [20]. Similarly, Valdez-Peña found the activity of Alcalase and trypsin for the deproteination of chitin [21]. Furthermore, protease secreted by several bacterial strains during fermentation in the presence of shrimp waste have been reported in many studies. Lee et al. reported the activity of *Paenibacillus elgii* TKU051 in deproteination once fermented on shrimp waste, where a maximum of 96.86% deproteination was obtained after seven days of fermentation [22]. Younes et al. showed the activity of crude protease resulted from the *Bacillus mojavensis* A21, *Balistes capriscus Bacillus licheniformis* NH1, *Bacillus licheniformis* MP1, *Vibrio metschnikovii* J1, *Aspergillus clavatus* ES1, where A21, A26, J1, and MP1 reported to have an activity of efficiency of deproteination of about 76 ± 4% and that of NH1 and ES1 were significantly lower, resulting in 65 ± 3% and 59 ± 3%, respectively [23,24]. Therefore, it is evident that deproteination of chitin could be achieved by treating with alkaline solutions, proteolytic enzymes, and proteases secreted during the fermentation of some microorganisms. Deproteinated chitin is then subjected to N-deacetylation, where the acetyl group is removed to produce chitosan. Deacetylation is commonly performed when deproteinated chitin is subjected to a treatment with a highly alkaline (40–50%) NaOH solution. The dielectric constant of NaOH is greater than that of KOH, making NaOH more suitable for the removal of the acetyl group. Chitin is composed of crystalline and amorphous regions, where the crystalline regions are reluctant to the deacetylation and complete amorphization increases the degree of deacetylation [25]. Deacetylation depends on the concentration of NaOH, reaction time, temperature, and the properties of the alkaline solution used. Furthermore, deacetylation of chitin can be achieved enzymatically when chitin deacetylase is used to remove the acetyl groups [26,27,28]; however, enzymatic deacetylation possesses disadvantages including high cost and low productivity, which limit the products to low molecular weight and amorphous chitin [8,28]. In addition to the main steps in converting chitin to chitosan, decoloration has also been used in many studies to remove the pigments such as astaxanthin and β-carotene associated with chitin using organic solvents including acetone, sodium hypochlorite, and hydrogen peroxide [16,29].

## 2. Synthesis of Chitosan Nanoparticles

**Figure 1:**
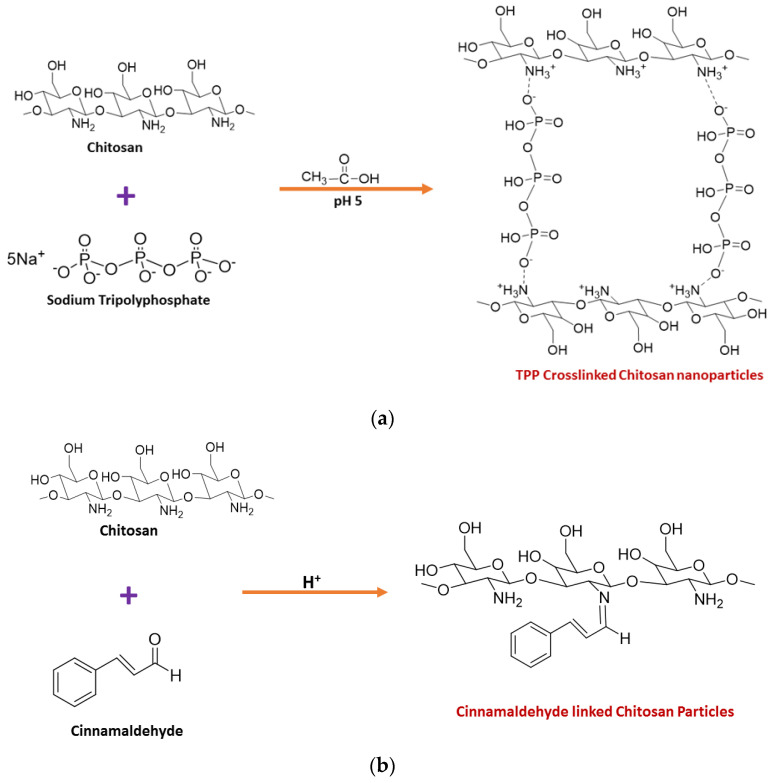

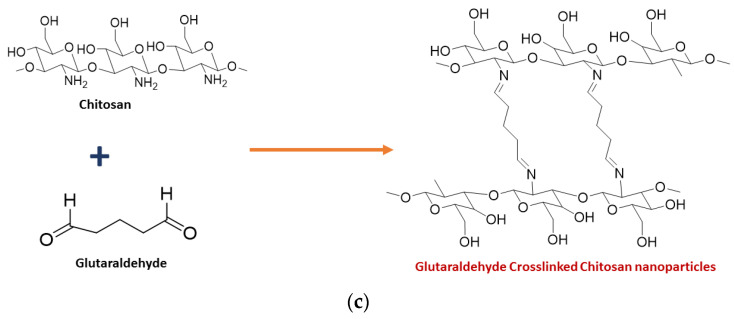
Synthesis of chitosan nanoparticles using (**a**) TPP (**b**) cinnamaldehyde (**c**) Glutaraldehyde as the crosslinker.

Chitosan nanoparticles have been synthesized by many methods, including ionic gelation [30,31,32], microemulsion [33,34,35], spray drying [36,37,38], and the reverse micellar method [39,40], of which the ionic gelation method has been found to be more promising due to the use of a cross linker. Low molecular weight chitosan was dissolved in 1.0% acetic acid, and the pH was then raised to 5.0 by adding 1 M NaOH solution. Sodium tripolyphosphate (TPP), the cross linker, was added, which forms electrostatic interactions between negatively charged TPP and positively charged chitosan. The obtained precipitate was centrifuged and washed to yield pure chitosan nanoparticles [31]. The synthesis of CNPs from the ionic gelation method using TPP is summarised in Figure 1a. The green synthesis of chitosan nanoparticles was also reported in several studies. El-Naggar et al., reported the biosynthesis of CNPs using *Pelargonium graveolens* leaf extract, which was effective in inhibiting the growth of the phytopathogenic fungi *Botrytis cinerea* [41]. Gadkari et al. studied the effect of cinnamaldehyde-cross-linked CNPs on inhibiting the growth of *Staphylococcus aureus* and *E. coli* where cinnamaldehyde was used as the cross-linker instead of TPP, as shown in Figure 1b [42]. Galan et al., reported the use of chitosan crosslinked with glutaraldehyde for the removal of reactive blue 4 dye [43]. Hence, it is evident that glutaraldehyde could also be used as a cross-linker to join chitosan molecules, as shown in Figure 1c.

Duraisamy et al., found the antibacterial properties of CNPs prepared in the presence of an ethanol extract of *Martynia annua* against *Bacteroides fragilis*, *Streptococcus oralis* MTCC 2696, *Propionibacterium acnes* MTCC 1951, *Pseudomonas aeruginosa* MTCC 424, *Staphylococcus aureus* MTCC 2940, *E. coli* MTCC 443, *Bacillus cereus* MTCC 441, *Streptococcus mutans* MTCC 890, *Aeromonas hydrophila* MTCC 12301, and *Streptococcus faecalis* [44]. The green synthesis of CNPs is further reported in other studies, which will be elaborated on with the applications in this review paper [45,46].

## 3. Importance of Crosslinking

**Figure 2:**
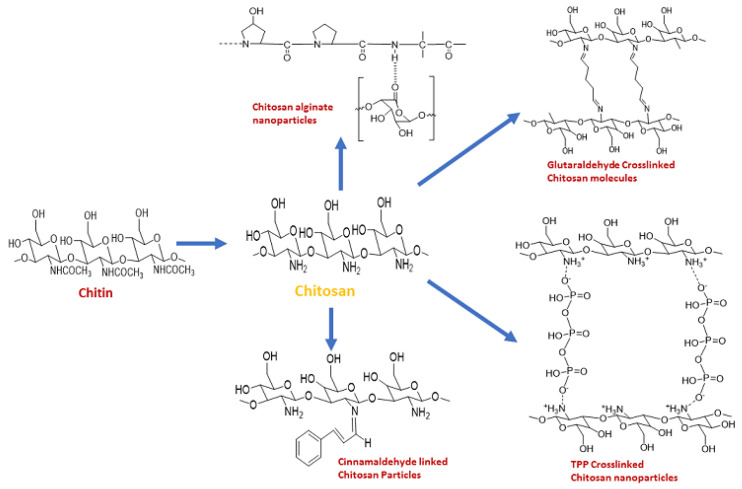
Different types of crosslinked chitosan.

Cross-linked chitosan, which is a modified version of chitosan, possesses different characteristics from native chitosan. Chitosan produced using different crosslinking agents is summarised in Figure 2. When a polymer is cross-linked, a permanent covalent network forms, which may permit the free diffusion of water and bioactive substances and also improve the mechanical properties of the polymer [47]. Chitin that has been cross-linked using an aldehyde/ amino group ratio of 1.0 has been used as an example to evaluate the impact of crosslinking on thermal performance [48]. In the following temperature range, 145–220 °C, the cross-linked films of chitosan have shown properties of expansion. Despite the fact that all chitin, partially deacetylated chitin, and chitosan do not exhibit this phenomenon, partially cross-linked water-soluble chitin did at 145 °C. The intermolecular hydrogen bonds formed by the polysaccharide molecules’ loose crosslinking are thought to account for this unique thermal behavior [48].

Results highlight the significance of hydrophilicity and suggest that it is essential to develop chitin derivatives that are highly hydrophilic while remaining insoluble in water in order to generate adsorbents with high capacity. A such asly and promising method to accomplish insolubilization without compromising the high hydrophilicity and the majority of amino groups is the loose crosslinking of the water-soluble chitin. Previous researchers showed that crosslinking was efficient in preventing the breakdown of chitosan in acidic liquids when used under heterogeneous conditions [48]. Chitosan’s functional properties can be improved by various crosslinking approaches. Since chitosan is more hydrophilic than chitin, it has the disadvantage of losing a significant amount of tensile strength when wet. It is clear that crosslinking increases the chitosan fiber’s strength, particularly its wet tenacity, according to a study previously conducted [49].

Chitosan possesses distinct chemical and biological properties, such as polycationicity, biocompatibility, biodegradability, hypocholesterolemic, anti-inflammatory, non-toxicity which renders the use of chitosan as a bioactive polysaccharide has been frequently used in several areas. The use of chitosan in biomedical applications, food biotechnology, wastewater management, agricultural applications, personal care, etc. is explained in detail in this review. Furthermore, the challenges associated with the use of chitosan in those applications are presented. Additionally, potential future applications are summarised in this review.

## 4. Applications of Chitosan

**Figure 3:**
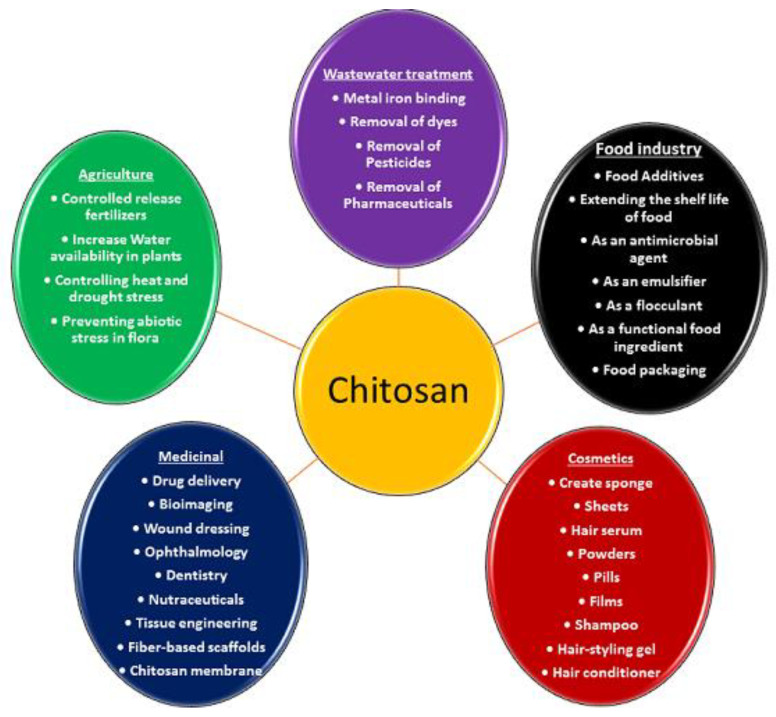
Applications of chitosan-based materials.

Chitosan is very well known for its wide variety of applications, including medicine, pharmaceuticals, food, cosmetics, agriculture, the textile and paper industries, the energy industry, and industrial sustainability, due to its unique biological and physiological properties, including solubility, biocompatibility, biodegradability, and reactivity. Applications of chitosan-based materials are summarised in Figure 3.

### 4.1. Drug Delivery

Drug administration is crucial in curing disease conditions, and an ideal drug is supposed to reach the disease tissue and accumulate at the correct concentration [50]. Among the drug administration routes, including oral, injection, transdermal, rectal, vaginal, and inhalation, oral administration has become more effective due to the ease of administration and the minimal side effects induced. Drugs can also be administered via injection, such as intravenous, intramuscular, intra-arterial, and subcutaneous, which creates a rapid response to the disease condition [51,52]. However, such administered drugs are subject to obstacles in the circulatory and digestive systems, and hence the proper chemical formula of the drug would not reach the disease tissue in a proper concentration [52,53]. The circulatory system and some organisms reject the drugs due to an immune response and tend to clear the system by degrading the drug molecules. Furthermore, drugs tend to hydrolyze and lose structural integrity in the acidic environment of the stomach [54]. Enzymes that are available especially in the gastrointestinal track as well as in the circulatory system and in organs biologically degrade drugs whose active component has a biological origin such as proteins and nucleic acids [55]. Therefore, it is essential to develop a proper drug delivery system to overcome the above-mentioned drawbacks, and chitosan has shown to be a suitable candidate because it is a biocompatible, biodegradable, bio renewable, non-toxic, and non-allergenic polymer [56,57,58]. More specifically, chitosan has shown to protect the drug molecules from the acidic environment in the stomach, adhere to the mucosal tissues to enhance the adsorption of specific drugs, ease in combining with the anionic drugs, and facilitate the colon administration [50,54].

#### 4.1.1. Drug Delivery to Proliferating Tissues

**Scheme 2:**
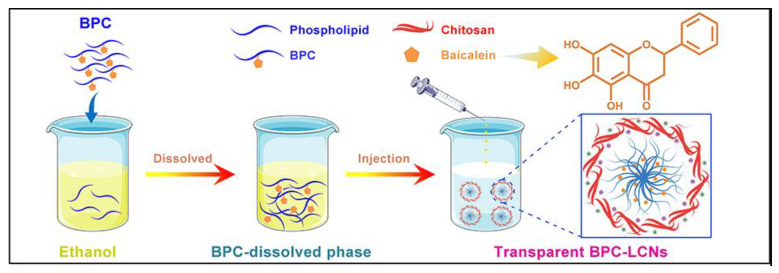
The preparation process of Baicalein-phospholipid loaded Lecithin/chitosan. Scheme adapted from Ref. [59].

Dong et al. [59] reported the use of Lecithin/chitosan nanoparticles to deliver Baicalein, a flavonoid that has multiple activities against dermatoses. However, applying Baicalein as a drug is limited due to its poor hydrophilicity and lipophilicity. Baicalein complexing with phospholipids improves the lipophilicity. The preparation process of Baicalein-phospholipid-loaded Lecithin/chitosan is shown in Scheme 2. Baicalein-phospholipid once entrapped in Lecithin/chitosan nanoparticles showed improved transdermal retention and permeability of Baicalein. Lecithin/chitosan nanoparticles encapsulating Baicalein-phospholipid showed superior colloidal stability, which helps to enhance the transdermal retention and permeability of Baicalein. Diameter, zeta potential, content, entrapment efficiency, and the appearance of the composite were hardly changed for three months, indicating higher stability of the drug encapsulated polymer. The promising colloidal and long-term stability have been ascribed to the cumulative effect of high zeta potential and high entrapment efficiency. Furthermore, the composite has shown to be effective in sustaining the release of the drug, which occurs due to the slower diffusion of Baicalein from Lecithin/chitosan which helps prevent the spreading of Baicalein over the stratum corneum. Moreover, positively charged Baicalein from Lecithin/chitosan is a product of negatively charged lecithin, and positively charged chitosan can interact with the negatively charged skin, facilitating prolonged retention and facilitating the penetration of nanoparticles across the skin barrier [59].

Fereig et al. [60] studied the effect of encapsulating the tracrolimus drug, which shows anti-proliferative action by T-lymphocytic cell inhibition, by chitosan nanoparticles of about 140.8 nm with an entrapment efficiency of 65.5%. Local skin deposition of the drug was enhanced with 82% of the drug retained in the skin compared with the existing tracrolimus topical treatment. The composite formula exhibited an enhanced hair growth rate, which reflects the skin recovery from the induced psoriatic plaques after the treatment [60]. Curcumin has attracted attention due to its pharmacological properties, including antimicrobial, neuroprotective, cardioprotective, and anticancer, but applying curcumin as a pharmaceutical has been limited due to its poor aqueous solubility and low bioavailability. However, curcumin-loaded Lecithin/chitosan nanoparticles entrapped 77.39 ± 1.70% of curcumin and have shown increased release of curcumin (86.18 ± 1.5%) compared with the curcumin release from the aqueous solution (14.81 ± 0.10%) in 24 h, and the release of curcumin by Lecithin/chitosan nanoparticles followed Korsmeyer–Peppas model with Fickian diffusion (n < 0.45). Furthermore, the curcumin- Lecithin/chitosan was physically stable, as confirmed by the insignificant changes in the particle size, polydispersity index, zeta potential, and %drug content monitored for 45 days [61]. Risperidone is a drug being used to treat schizophrenia is a highly hydrophobic drug that undergoes extensive hepatic metabolism, which leads to variations in its bioavailability. It is being administered either orally or via injection; however, orally administered drugs tend to undergo phase-I biotransformation, resulting in a lack of the ideal drug concentration reaching the target site. Resperidone loaded chitosan nanoparticles of particle size 86 nm with a polydispersity index of 0.287 and zeta potential of 36.6 mV showed an entrapment efficiency of 77.96 ± 1.50%. The micoadhesion efficiency of Risperidone loaded nanoparticles was observed to be 68.9%, leading to intranasal administration with better bioavailability. Chitosan nanoparticles released risperidone at about 90–100% which followed a biphasic controlled release with the Fickian diffusion mode. The drug and the delivery system were found to be physiochemically intact over a period of six months. Thus, it is evident that chitosan nanoparticles are an effective delivery system to deliver Risperidone [62]. Co delivery of curcumin and cisplatin from lipid-chitosan nanoparticles for enhanced cytotoxicity is reported by Khan et al. [63]. Optimised nanoparticles with a lipid to chitosan 20:1 ratio of about 225 nm showed more than 85% encapsulation efficiency. Controlled release of both curcumin and cisplatin was observed, but only 50% and 68% of curcumin and cisplatin, respectively, were released in 24 h. This results in the sustained release of drugs and avoids a higher therapeutic level. The lipid-chitosan nanoparticles are suitable for the delivery of both lipophilic and hydrophilic drugs, where the polymer provides higher encapsulation and prevents any rapid burst release of the drug. Furthermore, the lipid layer provides a diffusional barrier that enhances the controlled release of the encapsulated drugs. There was no significant enhancement in cytotoxicity of co-loaded hybrid nanoparticles compared with the cisplatin-loaded hybrid nanoparticles, but there was a significant increase in the cytotoxicity of the co-loaded hybrid nanoparticles compared with curcumin-loaded hybrid nanoparticles after 24 h. However, the cytotoxicity of co-loaded lipid-chitosan nanoparticles was greater than that of cisplatin and curcumin individually loaded hybrid nanoparticles because the addition of curcumin to cisplatin nanoparticles increased the chemo-sensitization of cells and resulted in higher cytotoxicity [63].

#### 4.1.2. Posterior Segment Ophthalmic Drug Delivery

**Figure 4:**
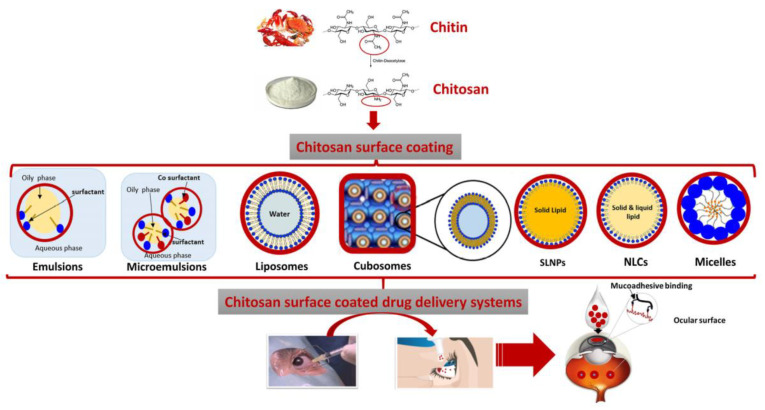
Use of chitosan surface coating on posterior segment ophthalmic delivery. Figure adapted from Ref. [64].

Age-related macular degeneration (AMD) and diabetic retinopathy (DR), two posterior segment eye disorders (PSEDs), are among the leading causes of permanent blindness globally. Due to the various obstacles, highly invasive intravitreal (IVT) injections are the main method used to deliver medications to the tissues of the posterior eye. Thus, the possibility of a topical delivery method that is more patient-friendly has been thoroughly researched. Precorneal clearance may be slowed while precorneal residency is extended by mucoadhesive formulations. As a result, they should increase the such aslihood of adhesion to corneal and conjunctival surfaces and hence provide enhanced distribution to the posterior eye segment. Due to its exceptional mucoadhesive properties, chitosan is the mucoadhesive polymer that has been studied the most [64]. Use of chitosan surface coating on posterior segment ophthalmic delivery is summarised in Figure 4.

**Figure 5:**
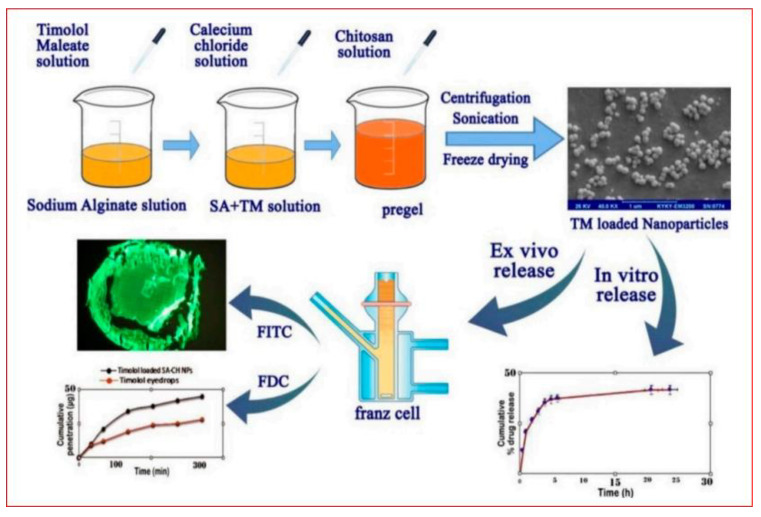
The synthesis of diclofenac sodium-loaded-N-trimethyl chitosan nanoparticles and application of them for ophthalmic usage. Figure adapted from Ref. [65].

Currently, only an eye drop solution of the ophthalmic preparation of diclofenac sodium (DC) for treating ocular inflammation is offered on the market. Limited patient compliance and quality of life result from the frequent application required by its low ocular bioavailability. In order to increase the ocular bioavailability of diclofenac sodium, this study was done to create formulations of diclofenac sodium-loaded N-trimethyl chitosan nanoparticles (DC-TMCNs) for ophthalmic usage. Using the ionic gelation method, diclofenac sodium-loaded N-trimethyl chitosan nanoparticles with various formulation compositions were created, and their physicochemical characteristics, drug release, potential for eye irritation, and ocular absorption of diclofenac sodium have been assessed. To produce diclofenac sodium-loaded N-trimethyl chitosan nanoparticles, N-trimethyl chitosan (TMC) was produced and quaternized to a degree of 49.8%. Depending on the amount of N-trimethyl chitosan and sodium tripolyphosphate present, the produced diclofenac sodium-loaded N-trimethyl chitosan nanoparticles had particle sizes between 130 and 190 nm, zeta potential values between +4 and +9 mV, and drug entrapment efficiencies of greater than 70%. The weight ratio of the N-trimethyl chitosan, diclofenac sodium, and tripolyphosphate in the optimised diclofenac sodium-loaded N-trimethyl chitosan nanoparticles formulation was 3:1:1. Their lyophilized product had a drug release pattern that matched the zero-order model after being reconstituted with phosphate buffer solution pH 5.5. The ophthalmic safety studies for diclofenac sodium-loaded N-trimethyl chitosan nanoparticles revealed that they were not harmful. The ophthalmic bioavailability of diclofenac sodium may be enhanced by diclofenac sodium-loaded N-trimethyl chitosan nanoparticles, according to an investigation on in vivo ophthalmic medication absorption. The findings of a certain study have suggested that diclofenac sodium-loaded N-trimethyl chitosan nanoparticles might be used as an alternative to traditional diclofenac sodium eye drops for the treatment of ocular inflammation [65]. Figure 5 summarizes the synthesis of diclofenac sodium-loaded N-trimethyl chitosan nanoparticles and their application for ophthalmic usage.

#### 4.1.3. Controlled Release Topical Ophthalmic Delivery

Montmorillonite/chitosan nanoparticles are a unique controlled-release topical ophthalmic delivery technique for the treatment of glaucoma. Since it has resulted in a short preocular residence period and low bioavailability, the quick clearance from the ocular surface has until now been a significant barrier for employing eye drops to treat glaucoma. The new nanoparticles were created by intercalating betaxolol hydrochloride (BH), a selective beta-adrenergic blocking agent, into the Na-montmorillonite (Na + Mt) interlayer gallery and then pursuing chitosan nanoparticles. The nanoparticles were designed for a topical, ophthalmic-controlled drug delivery system. The resultant nanoparticles had an average diameter of 460 nm, a positive charge of (+290.18 mV), and a positive charge. A regulated release pattern was indicated by an in vitro examination of the medication release characteristics. Both the chorioallantoic membrane-trypan blue staining (CAM-TBS) and human immortalized cornea epithelial cells (iHCEC) irritation experiment analyses revealed good tolerance for ocular tissues. Interesting results of the cellular uptake experiment determined by confocal laser scan microscopy (CLSM) revealed that the nanoparticles may get into human immortalized cornea epithelial cells.

In the meantime, BH-Mt/CS nanoparticles appeared to be able to extend the retention period in contrast to the BH solution in an in vivo study of the preocular retention capacity using multilayered human immortalized cornea epithelial cells to replicate the barrier of corneal epithelial cells. The micro dialysis sampling technique used to study the ocular pharmacokinetics revealed that the bioavailability of BH-Mt/CS NPs was higher than that of BH solution, with AUC0t and MRT0t being 1.75- and 1.99-fold higher, respectively. In addition, a study of blood drug concentration, on which only a few researchers have reported, revealed that low-level medications might enter the blood, indicating less severe overall side effects. BH-Mt/CS nanoparticles may significantly lower intraocular pressure in glaucomatous rabbits, according to important pharmacodynamics investigations. It has been anticipated that the BH-Mt/CS nanoparticles will be a promising carrier for BH, opening up the possibility of applications in the treatment of glaucoma. This is inspired by the development of montmorillonite/chitosan nanoparticles [66].

#### 4.1.4. Nanocarriers Based on Chitosan for Ophthalmic Use

Cationic polymer chitosan is extremely effective in delivering drugs, among other things. Because of its charge-based nature and mucoadhesive qualities, this polymer has frequently been investigated for drug delivery through the ocular route. It has been investigated to use chitosan and its various derivatives to deliver medicines, proteins, and peptides under regulated conditions. Due to its well-known safety issue for ocular distribution purposes as documented in the literature, it has been the formulation development scientist’s preferred option, either alone or in combination with other polymers/lipids/etc. [67]. How layer-by-layer deposition, based on natural polymers (chitosan and alginate), might be used to regulate the release of various ophthalmic drugs from three different types of lens materials has been studied: a silicone-based hydrogel that a certain group recently proposed as a drug-releasing soft contact lens (SCL) material and two commercially available materials, CI26Y for intraocular lenses (IOLs) and Definitive 50 for SCLs.

The optimised coating proved to have excellent features to control the release of the anti-inflammatory, diclofenac, while maintaining or improving the physical properties of the lenses. It is composed of one double layer of (alginate − CaCl_2_)/(chitosan + glyoxal)/(alginate − CaCl_2_), topped with a final alginate-CaCl_2_ layer to avoid chitosan degradation by tear fluid proteins. For at least a week, the coating causes a controlled release of diclofenac from soft contact lens and intraocular lens materials. Due to its great biocompatibility (water contact angle 0) and hydrophilicity (water contact angle 0), it shouldn’t require further surface treatments to increase user pleasure. However, because this coating’s barrier effect is unique to diclofenac, it is clear that the chemical makeup of the layers must be optimised with respect to the target medication [68].

The development of chitosan nanoparticles loaded with 5-fluorouracil (5-FU) for ocular delivery has been a main goal of study. Chitosan and a polyanion (Tripolyphosphate—TPP) have been used in an ionotropic gelation method to create chitosan nanoparticles. SEM and an atomic force microscope both revealed that the nanoparticles were uniform and spherical (AFM). The chitosan/TPP mass ratio and TPP had a substantial impact on the encapsulation effectiveness and particle size shape. The nanoparticle had a positive zeta potential of 304 mV and measured between 114 and 192 nm in size. The nanoparticle’s respective encapsulation effectiveness, loading capacity, and recovery were found to be 8.12–34.32%, 3.14–15.24%, and 24.22–67%. X-ray diffraction (XRD) and Fourier transform infrared (FT-IR) were used for physical characterization. The drug and polymer were not found to interact, and drug-loaded nanoparticles maintained their drug’s original crystallinity. In vitro research on nanoparticle release revealed diffusion-controlled release. In rabbit eyes, batch CS9’s bioavailability was investigated and compared with a solution of 5-FU. The rabbit eye’s aqueous humor had substantially more 5-FU than the rest of the eye. New Zealand rabbits’ eyes were used to study ocular tolerance; the formulation used was found to be non-irritating and showed no signs of inflammation [69].

### 4.2. Gene Delivery

Similar to drug delivery, chitosan has been effectively used for gene delivery as well. Abdelhamid et al. reported the synthesis and application of hierarchically mesoporous carbon nanomaterials derived from carbonized chitosan-encapsulated zeolitic imidazole frameworks (ZIF-8) for gene delivery. The obtained material has a high surface area (741 m^2^/g), a high mesopore volume (0.39 m^3^/g), and a large size (20–80 nm). Precisely they acted as a biocompatible non-viral vector for gene delivery using two oligonucleotides called Luciferase-expressing plasmid and splice correction oligonucleotides. Hierarchically mesoporous carbon nanomaterials enhanced the transfection efficiency of cell penetrating peptides (PF-14 and PF-221) by ten times due to the synergistic effect of hierarchically mesoporous carbon and cell penetrating peptides. The cell-penetrating peptides have been taken into cells through the scavenger receptor class A mechanism where the cell-penetrating peptides interact with scavenger receptor class A induces caveolae-mediated endocytosis, which consequently generates pores in the cell membrane for direct translocation [70]. Methyl methacrylate modified chitosan conjugates were prepared via the Michael addition reaction. An increased cell viability and transfection efficiency of the conjugates over pure chitosan were a result of the in vitro cytotoxicity and transfection experiments performed for all three cancer cell lines tested (A549, HeLa, and HepG2) because of the complexation of chitosan with negatively charged DNA. Furthermore, in vitro drug release study of the curcumin-loaded methyl methacrylate-modified chitosan conjugate nanoparticles showed that pH 5 is more desirable for the drug release than the physiological pH [71]. Insufficient intracellular gene release of chitosan has been overcome by reconstructing chitosan with propylamine, (diethylamino) propylamine and N, N-dimethyl-dipropylenetriamine which enhances the buffering capacity of chitosan. Furthermore, they enhance the gene binding and endosomal escape and N, N-dimethyl- dipropylenetriamine-modified chitosan has been shown to be effective in gene delivery, and the complex has been used to deliver the therapeutic p53 gene in A549 bearing mice, showing efficient therapeutic potential for cancer [72]. Furthermore, chitosan and its derivatives have been used for gene delivery in many studies for a variety of applications [73,74,75,76].

### 4.3. Bioimaging

Harish et al., reported the wet synthesis of chitosan-coated and uncoated CdS nanoparticles. Cytotoxicity activity of the uncoated and chitosan-coated CdS nanoparticles was determined in Human Jurkat and Human erythrocyte cell lines using the MTT assay. The percentage of viable cells was higher in all the CdS concentrations used in chitosan-coated CdS than in uncoated CdS nanoparticles in both Human Jurkat and Human erythrocyte cell lines, where the percentage of viable cells decreased with increasing CdS concentration in both chitosan-coated and uncoated nanoparticles. The cytocidal effect of CdS nanoparticles is caused by the release of Cd^2+^ ions to the medium, which bind with the sulfur, oxygen, and hydrogen atoms of the intracellular amino groups through covalent and ionic bonds, creating an imbalance in the homeostasis of the cells and leading to ROS generation. When CdS is coated with chitosan, the leaching of Cd^2+^ ions is reduced because the Cd^2+^ ions bind with the nitrogen atoms in the chitosan structure. The cellular internalization of the chitosan-coated CdS nanoparticles were determined in the Jurkat cell line. The chitosan-coated CdS nanoparticles entered the cells and fluoresced, making them easily detectable. Thus, it is evident that chitosan-coated CdS nanoparticles can be used as an efficient bio-imaging agent because of their lower toxicity [77].

Shi et al., studied the effect of the novel polymer based on aggregation-induced emission fluorogen, biotin, and disulfide bonds modified chitosan, which self-assemble into micelles in aqueous media and encapsulate paclitaxel into the core with higher drug loading. The emitted intense blue fluorescence indicated that the micelles showed excellent aggregation-induced emission features and could disassemble rapidly in the presence of high levels of glutathione. Modification by biotin increased the cellular uptake of the micelles, and they possessed high cytotoxicity against MCF-7 cells, where the distribution in the cells has been traced due to the aggregation-induced emission feature. Paclitaxel-loaded modified chitosan micelles exhibited anti-tumor activity, suggesting that they could be used for bioimaging and as a carrier for Paclitaxel [78]. Xu et al., also reported the encapsulation of paclitaxel into the micelles produced by the cetyl 4-formylbenzoate alkyl and 4-(2-hydroxyethoxy) benzophenonesalicylaldazide modified biotinylated chitosan, and they exhibited high aggregation-induced emission activity. Furthermore, they showed high drug release at acidic conditions along with selective internalization by MCF-7 cells and super cellular imaging ability [79].

### 4.4. Wound Dressing

Chitosan is derived from the naturally occurring chitin biopolymer and contains many desirable characteristics, such as biocompatibility and antimicrobial activity [80]. This renders chitosan highly suitable for wound dressing and aiding in the healing process. The application of chitosan in this aspect has been studied extensively in the recent past and has led to many advances. To act as an ideal wound dressing, these properties are vital, such as representing a physical barrier that is permeable to oxygen but at the same time maintains or provides a moist environment, is sterile and non-toxic and protective against microorganism infections, provides an appropriate tissue temperature to favour epidermal migration and promote angiogenesis, and is non-adherent to prevent traumatic removal after healing [81].

Wound dressings can be formulated into many forms, such as fibres, gels, membranes, films, sponges, and hydrocolloids. The term “film” and membrane can be used to describe the same type of dressing, although definitions differ according to the hydration as a hydrated film can be considered as a membrane [82]. The production of chitosan fibres has been recorded as early as the 1920s, while in the present, more advanced techniques such as electrospinning has been adopted in the production of chitosan fibres [83]. The synthesised N,N,N-trimethyl chitosan (TMC) fibres have different degrees of quaternization (DQ 19%, 25%, and 32%) containing a permanently positively charged ammonium group resulted in the increased antimicrobial activity of chitosan. This was due to the attachment of the positively charged molecule to the negatively charged cell membrane [84].

Hydrogels are another mode of applying chitosan as a wound dressing. These gels consist of a 3d polymer network that can absorb water. Hydrogels are moist, flexible, and soft, enabling applications in wound dressing. Hydrogels can function as carriers for drugs and growth factors, speeding up the healing process. Antimicrobial compounds can be covalently bound or non-covalently encapsulated in the hydrogel. to the addition of this inherent antimicrobial function of chitosan can function advantageously [85]. Chitosan membranes can be produced through solution casting- evaporation. Firstly, the chitosan was dissolved in a solution of dilute acetic acid and cast to produce a membrane. However, the presence of acetic acid and other crosslinkers such as carbodiimide or glutaraldehyde can interrupt the wound healing process due to their cytotoxic effects on mammalian cells. This disadvantage was overcome by using a chitosan floccule in the production of a chitosan-glycerol membrane loaded with antimicrobial agents by Ma et al. [86].

Chitosan films were also developed and incorporated with various anti-microbial agents. A chitosan film incorporated with *Hypericum perforatum* produced by Güneş & Tıhmınlıoğlu et al., showed increased antibacterial activity against *S. aureus* and *E. coli* [87]. In a study by Colobatiu et al., a quality by design approach was used to improve the process of producing bio-active compound-loaded chitosan films for wound dressings, enabling further understanding of the production process and optimization of the film formulation [88]. Nano silver particles were integrated into chitosan films to improve the antibacterial activity of the film. A potential wound dressing was created by the integration of SBA 15 supported nanosilver particles by Ambrogi et al., The films showed good hydration and strong anti-bacterial properties against both gram negative *Pseudomonas aeruginosa*) and gram positive (*Staphylococcus epidermidis* and *S. aureus*) bacteria [89].

Sponges are useful in the process of wound healing due to their ability to absorb large amounts of fluid. A non-leaching ampicillin grafted chitosan sponge was produced through chemical methods by Wu et al. [90]. This sponge showcased excellent antimicrobial activity against *Staphylococcus aureus*, *Candida albicans*, and *Escherichia coli* as well as speeding up the wound healing process. The sponge was non-leaching, and the stability of sponges was shown to be excellent due to their insolubility and non-degradability after 14 days of immersion in PBS buffer. Hydrocolloids are moisture-retentive dressings that contain gel-forming agents such as sodium carboxymethylcellulose and gelatin [91]. Hiranpattanakul et al. develop a chitin/chitosan hydrocolloid (CCH) wound dressing. Chitosan was cross-linked with tripolyphosphate to prepare chitosan microbeads and then incorporated within the chitin matrix in chitosan microbeads: chitin (*w/w*) different ratios. The hydrogels were evaluated for their water absorption, enzymatic degradation, antibacterial activity against *Staphylococcus aureus* and *Escherichia coli*, and biocompatibility with the L929 cell line. These chitin hydrogels showcased higher water absorption compared with chitosan microbead: chitin hydrogel, and anti-microbial activity was apparent in both hydrogels, suggesting applications in the medical field [92].

Chitosan-based hydrocolloids are clinically proven to have a positive impact on wound healing. An efficacy study conducted by Liu and Shen showed that in a study group with patients having chronic refractory wounds, there was significant improvement after the use of chitosan hydrocolloids compared with the control group. The improvement was scored based on the wound healing efficiency, itching pain score, changes in the wound area, dressing change frequency, and cost after a 3 week period [93].

Several chitosan-based products are commercially available on the market for wound healing and controlling trauma. These products have been mainly focused on bleeding management and dressing wounds. Commercially available chitosan-related wound dressing products are summarised in Table 1.

### 4.5. Tissue Engineering Applications of Chitosan

Tissue engineering is an advanced area of reparative medicine that emerged from the field of biomaterials development. Basically, this technique repairs, improves, and maintains the function of injured tissue or organs by combining cells, biologically active molecules, and scaffolds [102]. Various forms of chitosan and chitosan-integrated scaffolds are used in tissue engineering, such as mesoporous scaffolds, hydrogels, fibre scaffolds, and microspheres. A more recent advancement utilizes 3d printing to produce scaffolds. These scaffolds can be used in the tissue engineering of various types of tissues, such as bone, cartilage, and skin. Natural bone tissue is composed of calcium-deficient carbonated hydroxyapatite as the inorganic phase and collagen type I as the main organic phase. The biomimetic approach to scaffold development for bone tissue engineering applications is focused on mimicking complex bone characteristics [103]. Mesoporous scaffolds are commonly created by phase separation followed by lyophilization. As the common solvent used in the dissolution of acetic acid, this should be neutralised to prevent dissolution of the scaffold in aqueous media [104]. Freeze gelation can be utilised as well for a similar outcome, where the scaffolds are placed in a gelation solution of sodium hydroxide and ethanol below the chitosan freezing temperature. Following the gelation, scaffolds are washed with ethanol and lyophilized [105]. A porogen can be utilised in combination with the above methods to increase the porosity. The porogens should be added to the chitosan solution prior to gelation and leached out after the formation of the scaffold. As well as this high-pressure CO_2_ can be used in the foaming method to produce desirable results [106].

#### 4.5.1. Fibre Based Scaffolds

Fibre-based scaffolds are formed through the process of electrospinning [107]. Electric fields are utilised in this process to regulate the deposition of fibres in order to form a scaffold. In comparison to synthetic fibres, natural fibres are harder to produce through spinning due to their limited solubility in most organic solvents, high molecular weight, polycationic character in solution, and three-dimensional networks of strong hydrogen bonds [108]. This was resolved in chitosan through an alkali treatment to hydrolyse chitosan followed by dissolution 70–90% acetic acid to produce nanofibers [109]. A fibrous mat of chitosan blended with polycaprolactone (PCL) was prepared by Prasad et al. for potential applications in wound healing. The results indicated that fibres supported adhesion and spreading of cells on their surface and that the cells were impregnated in the fibres as well. This chitosan fibre may serve as a biological substitute in skin tissue engineering. Nanofibers of chitosan coated with polylactic acid-collagen-aloe vera have been investigated for potential applications in skin tissue engineering. The synthesized chitosan-polylactic acid-collagen nanofibers possessed 67.5% porosity and a higher water uptake rate in comparison to the control polylactic acid-collagen. It was revealed that the nanofibers provided an optimal environment for cell proliferation through a cell culture assay. Similarly, the fibroblast attachment of a nanofiber-based scaffold of chitosan-polyvinyl alcohol was explored in a study by Koosha et al. [110]. It was revealed that the scaffold was biocompatible and enhanced cell proliferation, making it a candidate for tissue engineering. Another study exploring the development of a wound dressing with nanofibres of chitosan and polyvinyl alcohol with h carboxymethyl chitosan nanoparticles found that nanofibers effectively improved the biological activity of bioactive compounds, bioavailability, and further controlled the degradation. The nanofibers showed antimicrobial activity against *S. aureus* and *E. coli*, and chitosan-based nanofiber had improved collagen deposition and re-epithelialization pattern revealed through a histopathological assay of a wound model [111].

#### 4.5.2. Hydrogels

Preformed scaffolds give rise to various problems such as surgical implantation, increasing the risk of infection, and improper scaffold shape and size [88]. Injectable hydrogels can be used to overcome these obstacles [112]. Ideal hydrogels should remain liquid at room temperature while forming a gel when injected into the fractured location, filling the complex shape of the defect. The hydrogels should be able to shorten the length of the operation, reduce pain, lessen scarring, and minimize damage to the surrounding muscles [113]. Chitosan hydrogels are ideal for this application due to their solubility in mildly acidic environments; upon neutralization, a hydrogel can be formed. This is due to the removal of electrostatic interactions, which allows the amino group to form hydrogen bonds [114]. In situ synthesized hydroxyapatite in a chitosan solution (10 °C) was used to obtain a pH-responsive hydrogel at 37 °C. A slightly acidic environment in the prepared composite solution favours NaHCO_3_ dissociation, which releases HCO_3_^−^ ions responsible for carbon dioxide production and increases pH [115]. In skin tissue engineering, hydrogels are used as scaffolds for cell growth. The mechanical properties of chitosan have been improved by the addition of various cross-linking agents. In a study, chitosan hydrogel crosslinked with glutaraldehyde and genipin was prepared. This hydrogel contained a high porosity level with an average size of 60–80 μm, where the presence of a crosslinking agent maintained the porosity of the chitosan hydrogel. The cell growth capacity of the hydrogel was analysed by human skin fibroblast cells GM3348. An in-vitro cell proliferation assay confirmed a significantly higher cell proliferation in comparison to the control, while a histological analysis demonstrated that cells also penetrated inside the scaffold, showing an increased number of fibroblast cells at day 7 [116]. A novel hydrogel fabricated with chitosan and oxygenated fluorinated methacrylamide was tested for regenerative properties. The novel hydrogel not only increased reepithelization, increased collagen content, and neovascularisation but was also reported to have supplied oxygen to the diabetic mouse wound area [117].

#### 4.5.3. Chitosan Microspheres

**Figure 6:**
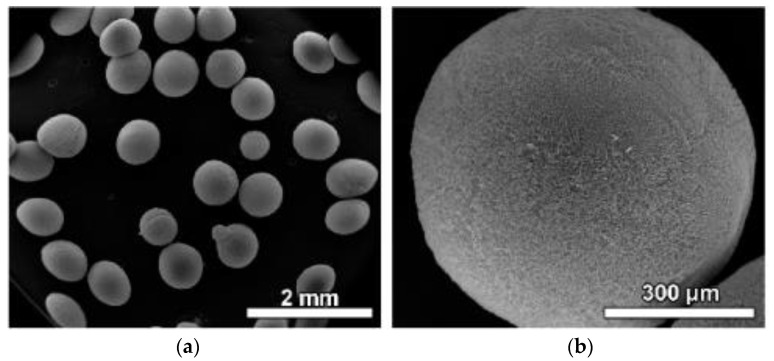
SEM images of chitosan microspheres at (**a**) low and (**b**) high magnifications. Figure adapted from Ref. [121].

Due to their biocompatibility and biodegradability, chitosan microsphere systems have been proposed for use as injectable bone-filling (non-load-bearing) biomaterials or drug delivery matrices [118]. These microspheres offer the same advantages as hydrogels, such as injectability and minimal surgical intervention (Fang et al.). Chitosan degradation products are not toxic for cells or the human organism; however, to obtain stable chitosan microspheres, chemical cross-linking is required to cross-link amino groups in the chitosan chain. Residual cross-linking agents in microspheres might have a toxic effect on cells, surrounding tissue, and the human organism. Complete removal of unreacted cross-linking agents from obtained scaffolds remains a challenge for Fang et al. Therefore, studies have been conducted to utilize nontoxic crosslinking agents in order to produce chitosan microspheres [119]. Highly porous chitosan microspheres were prepared through an emulsion-based thermally induced phase method with an average diameter of microspheres of ~150 μm and with interconnected pores in the range of 20–50 μm [120]. Obtained microspheres showed excellent biocompatibility with multidirectional cell–cell interactions (Huang et al.) due to the lesser bone adhesion capacity, pure chitosan microspheres cannot be used alone, and integration of other components such as hydroxy apatite will increase the adhesion capacity. As well as this, the chitosan microspheres (Figure 6) can be used as a filler compound for scaffolds formed from other materials. Furthermore, anti-cancer drugs such as 5-fluorouracil, paclitaxel, and cis-dichlorodiammine-platinum-eluted chitosan microspheres have been reported to be used for osteosarcoma and to significantly inhibit the growth and migration of both HOS and MG-63 cells [121].

#### 4.5.4. Chitosan Membrane

Membrane scaffolds can be used in skin tissue engineering, and to determine the capabilities of chitosan-based membranous structures for tissue engineering, their potential has been thoroughly investigated. A study created a chitosan coating with titanium dioxide nanoparticles that may have potential for structural and functional regeneration. The membrane construct was flexible, had good crystallinity, and had good mechanical properties. Antibacterial activity against *Staphylococcus aureus* was demonstrated using membranes. In comparison to the control group, which had a plastic surface, the application of chitosan membrane to mouse fibroblast L929 cells showed rapid proliferation, decreased oxidative stress, and apoptosis. Additionally, protein expression analysis demonstrated the presence of fibroblast-associated markers on the membrane surface of L929 cells, which are necessary for their survival and expansion [122]. Chitosan membranes loaded with glycerol and anti-microbial agents as well as membranes loaded with curcumin and *Aloe vera* extracts showed antimicrobial activity and cell proliferation of fibroblasts (Ma et al.). Another study investigated the anti-inflammatory properties of the chitosan-hyaluronan-edaravone membrane during wound healing tests. An in-vitro antioxidant test revealed that the membrane’s ability to scavenge free radicals was improved with the addition of edaravone. Additionally, in-vivo studies on the skin of rats showed that membranes promoted fibroblast, keratinocyte, and endothelial cell migration and suppressed the inflammatory response, hence promoting effective wound healing [123]. A different study produced chitosan film using agarose polymer. The resulting membrane had a pH of 5.98, which was similar to the skin’s pH. The composite membrane was also highly exudate absorbent and deformable elastic. In a stimulated enzymatic environment, the membrane also showed sensitivity, demonstrating the biodegradation of the film at the site of the wound and assisting in the release of active molecules for the healing process [124].

### 4.6. Dentistry

Chitosan has been widely applied in biodental applications due to its unique properties, including bioactivity, biocompatibility, and antimicrobial properties. Javed et al., studied the effect of amalgamating CuO-chitosan nanoparticles into dentine bonding agents, which showed success as a remedy against secondary caries. Furthermore, CuO-chitosan nanoparticles incorporated into dental adhesive discs produced a reduction in *Lactobacillus acidophillus* and *Streptococcus mutans*. Moreover, the coupled nanoparticles have shown to increase the mechanical features, water sorption, and slight change in shear bond strength, making them more applicable to dentistry-based applications [125]. Zeza et al. reported the use of a brush made out of chitosan to treat patients who were diagnosed with mild peri-implantitis. Modified bleeding index and probing depth were significantly reduced in two weeks compared with the baseline, and 73% of the patients showed no bleeding on probing with stable bone level at 24 weeks, indicating that a chitosan brush could be successfully applied for plaque removal and resolution of clinical signs of the initial stages of peri-implant inflammation [126]. Khan et al., similarly, reported the use of an oscillating chitosan brush to treat mild to moderate peri-implantitis, where elimination of the disease was found in 9.5% of cases in the group treated with an oscillating chitosan brush, compared with the 5.9% of cases in the group treated with a titanium curette [127]. Wohlfahrt et al. also found that the implants treated with the chitosan brush showed an improvement in bleeding on probing at 2 and 4 weeks compared with the implants treated with titanium curettes [128].

### 4.7. Ophthalmology

#### 4.7.1. Evaluation of Precorneal Retention and Tolerance

The mucoadhesive polysaccharide chitosan has been researched as a potential constituent in ophthalmic gels for prolonged precorneal drug residency lengths. This cationic vehicle was expected to thicken the solution and interact with the negative charges of the mucus to impede the lachrymal flow, which is the process by which medicines are removed from the body. Together with the molecular weight and concentration of polysaccharides in chitosan, the length of the precorneal residence time, and the ocular tolerability of formulations containing tobramycin as a therapeutic agent, is impressive. A covalently cross-linked chitosan hydrogel sheet was created for a different study and evaluated as an ocular carrier for the topical delivery of necessary medications. Here, glycolic chitosan has been utilized. The hydrogels’ gelation duration and moduli could be tailored by varying the component concentrations, according to rheological characterization. The proposed hydrogel’s non-irritant nature after topical applications has been proven by the ocular irritation tests. Covalently cross-linked chitosan hydrogel sheets may be used for topical ophthalmic medication administration because of these properties [129].

An eye irritation test using confocal laser scanning ophthalmoscopy and corneal fluorescein staining amply demonstrated the high tolerance of chitosan after topical application onto the corneal surface. Gamma scintigraph results showed that the clearance of the formulations labeled with 99mTc-DTPA was significantly delayed in the presence of chitosan compared with the commercial collyrium, regardless of the quantity and molecular weight of chitosan in the solution. When chitosan was present, the corneal residence time rose by at least three times. Since they offer the benefits of solutions such as accuracy and reproducibility of dosing or convenience of administration with an extended contact period of ointments, ocular in situ gels are a viable alternative to overcome the disadvantages of conventional eye drops. Chitosan is a natural polymer that is appropriate for use in ophthalmic formulations because of its above-mentioned qualities, including impacts on ocular wound healing and permeability augmentation. Due to the prolonged ocular contact period, the combination of chitosan, a pH-sensitive polymer, and other stimuli-responsive polymers increases the mechanical strength of formulations [130].

In order to create hydrogel-like ophthalmic drug delivery applications, flurbiprofen (an anti-inflammatory drug) and timolol maleate (an anti-glaucoma drug) were impregnated into three chitosan derivatives (N-carboxymethyl chitosan, N-carboxybutyl chitosan, and N-succinyl chitosan). This was done using the supercritical solvent SEM and FTIR spectroscopy were used to analyze the developed polymeric drug delivery devices as well as additional polymeric samples treated in CO_2_. For each created system, drug release kinetics investigations have been carried out. In comparison to the conventional soaking impregnation method, the effects of impregnation pressure and temperature on the outcomes of the release kinetics have been investigated. Results shown that the three distinct (chemically and physically) polymeric architectures influenced the impregnation and drug release processes under the same operational settings. Results have also shown that, for N-carboxymethyl chitosan, the predominant effects in the impregnation process seemed to be the solubility of drugs in CO_2_ and in CO_2_ + EtOH mixtures, as well as the swelling and plasticizing of the polymer. This is despite the fact that the final released drug mass is always the result of the employed operational impregnation conditions and of the very complex relative specific interactions that may occur between all species present in the system. The traditional impregnation of pharmaceuticals by a soaking method was shown to be less effective and “tunable” than the SSI method. Therefore, these N-chitosan derivatives-based ocular drug delivery systems can be quickly and effectively manufactured to take into account the necessary drug levels in accordance with patients’ needs by adopting this “tunable” SSI approach [131].

#### 4.7.2. Translational Ophthalmic Applications

Drug administration to the eye’s anterior and posterior parts is still found to be difficult. Drug delivery using nanoparticles has shown some potential. Chitosan-based monotherapies for ocular drug delivery are in the current development stage, and certain difficulties have also been faced since they deal with sensitive areas of living beings. Chitosan, a cationic linear polymer, has been extensively studied for ocular medication administration. Numerous studies have made use of the polymer’s mucoadhesive qualities, which result from interactions between the amino acids in chitosan and the sialic acid residues in mucous. The significant increase in ocular medication retention made possible by the high degree of crosslinking in chitosan nanoparticles aids in ocular penetration and increases the bioavailability of the pharmaceuticals. The development of a sustained drug release formulation using a biodegradable and biocompatible chitosan polymer was motivated by a noticeable reduction in the first burst of the medication. Studies conducted both in vitro and in vivo have revealed improvements in the absorption, accumulation, and clearance of chitosan nanoparticles from the delivery site. Numerous studies are being conducted on chitosan- or modified-chitosan-based nanoparticles as drug carriers for the treatment of bacterial and viral infections, glaucoma, age-related macular degeneration, and diabetic retinopathy [132]. A summary of using chitosan for ophthalmology is given in Table 2.

### 4.8. Chitosan as Nutraceuticals

The bioavailability and durability of bioactive food ingredients can be greatly improved by nanoencapsulation, a cutting-edge method that uses nanostructures. Chitosan has been used to create a variety of nanostructures, including nanoparticles, nanohydrogels, nanofibers, and nanocomposites, which have been successfully used as nanocarriers for encapsulating a variety of bioactive compounds, such as phenolic compounds, essential oils, carotenoids, and vitamins [136]. It has been discovered that although lipid targets may be reached, a significant residual cardiovascular risk still exists and that lipid-lowering medications may have unfavorable side effects. It has been demonstrated that treatment using substances that resemble bodily proteins, such as incretin-based medicines, holds potential for this particular purpose. However, lifestyle changes continue to be crucial for lowering cholesterol levels and reducing cardiovascular risk. Some studies have concentrated on nutraceuticals that could have a positive impact on metabolic parameters and lower cardiovascular risk. Dietary fiber chitosan helps control lipid levels and improve anthropometric measurements. To verify such advantages, larger, prospective clinical trials are needed. Such therapies may be suggested when lipid-lowering medications are neither warranted nor well-tolerated, as well as to meet therapeutic goals and/or reduce lingering cardiovascular risk [137]. Chitosan is the most prevalent marine mucopolysaccharide. Since its extraordinary biological activities have been studied and documented in order to take advantage of its nutraceutical features, chitosan can be regarded as a potential marine nutraceutical. The manufacture of biomedical materials as well as the functionalization of other polymeric materials, including fibers and food preservation, use chitosan’s antimicrobial activity, which has been regarded as its most important and influential bioactivity. Chitosan and its derivatives have been shown to have broad-spectrum antibacterial properties, according to a group of experts. They have established that chitosan can stop some microorganisms, such as bacteria, yeast, and fungi, from growing [138]. Despite extensive research to date, the mechanism behind chitosan’s antibacterial effect is still not entirely known. Since chitosan has more amine groups than chitin, which gives it its cationic properties, it has a significantly stronger antibacterial impact. However, chitosan was shown by numerous researchers to be a more effective inhibitor against gram-positive than gram-negative germs in their experimental findings. Scientists now find the free radical response to be a very fascinating topic because it is thought to be the primary cause of a number of distinct human ailments. Free radicals are highly reactive and unstable due to their atomic or molecule structure. As a result, in order to be in a more stable condition, they frequently pair up with other molecules and atoms. Chitosan has demonstrated a noteworthy scavenging ability against several radical species, opening up a wide range of potential applications. According to many ideas, the free radical-scavenging ability of chitosan derivatives depends on hydrogen atom donations [139].

**Figure 7:**
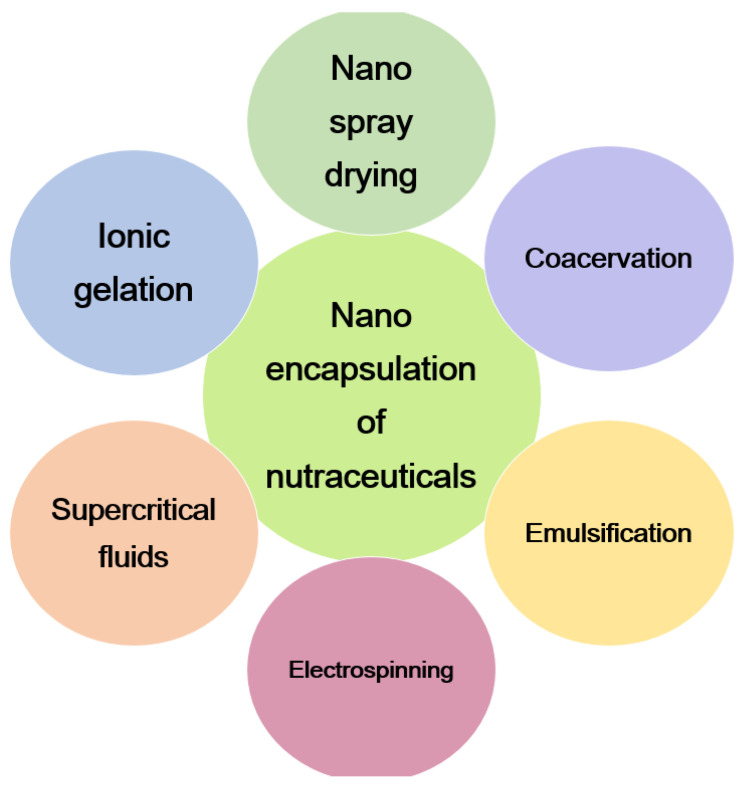
Nanoencapsulation of nutraceuticals with chitosan and its derivatives.

The life expectancy of patients has significantly increased as a result of clinically executed general cancer therapies employing chemotherapy, radiation, and surgery. The pharmacological characteristics of many current anti-cancer medications are not optimal, including low aqueous solubility, irritation, instability, rapid metabolism, and nonselective drug distribution. As a result, these medications can have a variety of negative effects, including subpar therapeutic activity, dose-limiting side effects, and poor patient quality of life. As a result, numerous scientists are motivated to look for safer and more effective treatments for cancer patients. The natural polysaccharide chitosan and its derivatives are thought to have anticancer properties. An increase in interest in polysaccharides can be attributed to several efforts to find an effective anticancer drug from natural sources. The goal of inflammation is to restore a tissue compartment’s structural and functional integrity following exposure to an aversive stimulus. Inflammation is the body’s initial protective response to infection or injury. The anti-inflammatory and pro-inflammatory effects of chitosan and its derivatives have been the subject of several studies [140]. Given the need for food product preservation and the rising concern over the harmful environmental effects of conventional packaging materials, biologically active molecules such as chitosan and its derivatives have a lot of potential in the food business. A biopolymer derived from chitin, chitosan is made from the byproducts of commercial fishing. This compound has a wide range of uses because of its distinctive chemical structure, which includes a linear polycationic chain with high charge density, reactive hydroxyl and amino groups, as well as substantial hydrogen bonding. Chitosan nanoparticles are a new food additive that is created using eco-friendly technology. By taking into account differences in the polymer’s molecular weight and level of deacetylation, the usefulness of these chitosan nanoparticles as a novel and organic antibacterial agent has been examined in several research projects [141]. Nanoencapsulation of nutraceuticals with chitosan and its derivatives is shown in Figure 7.

### 4.9. Cosmetics and Cosmeceuticals

Aqueous solutions or solid forms of chitosan and its derivatives, such as chitosan powder, can be used to enhance the effects of other hydrating agents, solar filters, and other bioactive items. Since chitosan is biocompatible, it can be used on the skin and is compatible with a variety of different substances, including glucose, oils, fats, and acids. It is a very potent hydrating agent with the ability to create films, providing water while preventing dehydration. Chitosan has multiple uses and can significantly improve personal care products by substituting unwanted substances and improving the permeability of other active components. Since it helps to retain skin hydration, tone skin, treat acne, offer extracellular matrix support, and encourage the skin’s natural barrier function, chitosan is frequently utilized in cosmetic and skin care products. Chitosan is a good component for anti-aging skincare products and wound healing since it is a natural stimulant in the processes of skin regeneration and wound healing, encouraging proper histoarchitectural tissue organization with optimal collagen structure.

In order to meet customer demands and international standards, the cosmetics business must design and develop new eco-sustainable products. For this, new substances with greener profiles must be used in place of conventional ones that come from petrochemical sources. However, this shift to using green components in the cosmetics sector cannot jeopardize the efficacy of the resulting goods. Chitosan and its derivatives, which combine a variety of intriguing physicochemical and biological qualities for the creation of cosmetic goods, are emerging ingredients in this new direction of the cosmetic business. The usage of chitosan thus paves the way for a potential future in the development of cosmetic formulations. Chitosan is a particularly good choice for the design of skin and hair care formulations due to its electrostatic interaction capabilities with negatively charged substrates (such as skin or damaged hair), which result in the formation of polymeric films that help to condition and moisturize cosmetic substrates [142].

The biologically active compounds found in marine resources have a wide range of potential uses in the cosmeceutical sector. The cosmeceutical industry is particularly interested in chitin and its deacetylated derivative, chitosan, among the several chemicals of marine origin because of their distinct biological and technological characteristics. Chitosan serves a variety of functional purposes, including as an oral hygiene aid, an ingredient in skin and hair care products, and a transporter for active substances. The only polycation found in nature, chitosan’s charge density is influenced by the medium’s pH and level of deacetylation. The level of deacetylation and molecular weight affect how soluble the polymer is. Chitosan oligomers are soluble at a variety of pH levels, including basic and acidic ones [143].

Application areas for chitin and chitosan include biomedicine, food additives, cosmetics, and more. Due to its proven antibacterial characteristics, the charged chitosan polymer is highly useful in biomedical applications. To expand the application fields in a number of domains, chitosan’s and its products’ various biological activities have been thoroughly studied. The solubility of water and other solvents has a significant impact on the natural characteristics of chitosan. Because they have higher water solubility and lower viscosity than chitosan, chitosan oligosaccharides with low polymerization degrees are receiving a lot of attention in the pharmaceutical and medical industries. The bioactivity of chitosan is influenced by the effects of chitosan on physicochemical factors, including molecular weight and deacetylation level [144].

Collagen, chitosan, and hyaluronic acid-based thin films have been produced, and their surface and mechanical characteristics have been investigated. Utilizing FTIR spectroscopy, contact angle measurement, and AFM pictures, the films’ structural makeup has been investigated. Additionally, mechanical and swelling analyses have been carried out. Collagen, chitosan, and hyaluronic acid have been researched for their potential to protect hair using SEM microscopy and mechanical testing of hair coated with the compounds. It has been discovered that hyaluronic acid boosts the mechanical resilience of biopolymeric films when combined with a collagen/chitosan mixture. Hyaluronic acid-infused samples had increased surface roughness and were more stable in aqueous environments [145].

#### 4.9.1. Cosmetic and Pharmaceutical Uses of Chitosan

Chitosan and its derivatives have undergone testing for application in the cosmetic and pharmaceutical industries in many projects. Chitin and chitosan have been used to create sponge sheets, beads, powders, pills, and films. The degree of substitution and the structure of the acyl groups in certain water-soluble and enzymatically digestible derivatives of chitin and chitosan were altered, and this in turn influenced the rate of their enzymic breakdown. These derivatives’ biodegradable, biocompatible, viscosity, and moisture-holding qualities make them suitable as cosmetic components. Chitosan-containing hair care products, chitin sutures that are absorbable in tissues, and chitosan-containing skin care products are a few examples of commercial goods [146].

#### 4.9.2. Chitosan Hybrids for Cosmetic Uses

Materials known as cosmeceuticals can benefit from both pharmaceuticals and cosmetics. Despite the fact that a variety of materials are employed, those made of biopolymers are the most important due to their superior biocompatibility and usefulness. Chitosan has been extensively studied as a natural biopolymer, among many others, and it has found use in a variety of applications, including tissue engineering and drug delivery. In addition to chitosan, its derivatives have made outstanding strides in the study of skin, hair, and dental care. The drug-loaded chitosan micro- and nanoparticles for diverse cosmeceutical applications have recently undergone substantial advances [147]. So far, only a few direct studies on the use of chitosan and its derivatives in cosmetic applications have been conducted. Due to their biological activity and potential uses in the sectors of medicine, food, pharmaceuticals, and cosmetics, chitosan and its derivatives have garnered a lot of interest. Due to their physicochemical and biological activity, as well as their typical bio-compatibility, biodegradability, and non-toxicity, these substances may be prospective agents in the cosmetic sector. Chitosan and its derivatives may make excellent candidates for sunblock, skin, oral, and hair care formulations because of their improved biological activities [148]. Commercially available chitosan included cosmetic products are given in Table 3.

### 4.10. Use of Chitosan-Based Derivatives in Food Industry

#### 4.10.1. Food Additives

Chitosan is highly pursued by the food industry due to its distinctive biocompatibility, biodegradability, bio-renewability, less-toxic nature, physiological inertness, and hydrophilicity [168]. Chitosan and their oligomers and monomers, which are derivatives of chitin, the first polysaccharide identified by humankind, have a wide range of physiological functions and applications related to the food industry [169]. In particular, chitosan and its oligomers and monomers are heavily exploited as food additives to enhance the flavor, improve the appearance, and extend the shelf life in the form of a preservative [170]. Apart from these, chitosan is used as a stabilizing agent, emulsifying agent, thickening agent, fining agent, antibacterial agent, mimic for low-calorie food, and antioxidant [169].

For better utilization of chitosan, it has been modified through several methods such as hydrolysis by chemical, enzymatic, and physical methods in order to achieve a reduced molecular weight to match the requirements of the food industry [171]. Chitosan has also been conjugated with various substances and compounds for improved biophysical and biochemical properties to enhance its potential as a food additive [172]. The cationic nature of chitosan related to the amino group at C-2 is promising as to be employed as a crosslinking agent, and this distinct property of chitosan linked with nanotechnology has paved the way to form nano-scale chitosan compounds with unique biophysical and biochemical properties [171]. Unique properties of nanoparticles such as solubility, diffusivity, less toxicity, colour, magnetic, optical, and thermo-dynamic properties due to their high surface ratio have further improved the potential of chitosan to be used as an additive in the food industry [173].

#### 4.10.2. Additive against Obesity

Obesity has been identified as a serious threat to human health in medicine and pharmacology, in the recent years. Overweight and obesity have been linked to an increased risk of developing chronic conditions such as coronary heart disease, type 2 diabetes, hypertension, stroke, dyslipidemia, insulin resistance, gestational diabetes mellitus, metabolic syndrome, and cancers of the breast, endometrium, prostate, and colon, according to epidemiological research. Numerous studies have been carried out on dietary supplements that induce weight loss and a reduction in body fat mass. Due to its ability to withstand digestive enzymes and acidic gastric conditions, chitosan is regarded as a source of dietary fiber [168]. Dietary fiber is considered to contribute no calories to human diet, nevertheless the metabolites produced by the bacteria in the colon are consumed by mammals to meet their energy requirements. Chitosan restricts the absorption of dietary cholesterol and the circulation of cholic acid to the liver after ingestion by forming micelles with cholesterol and dietary cholesterol in the alkaline fluids in the upper portion of the colon. Cholic acid is produced from blood cholesterol in the liver leading to decreased blood cholesterol concentration [174]. The intestinal microorganisms in the large intestine secrete chitinases which can digest the micelles and bile acids and sterols are excreted into feces without absorption [175]. A dietary fiber’s ability to decrease cholesterol depends on its high viscosity, polymeric structure, high water-binding capacity, non-digestibility in the upper gastrointestinal tract, and low water-binding capacity in the lower gastrointestinal tract [172]. Chitosan satisfies the majority of these requirements and differs from other dietary plant fibers in that it may bind anions such as free fatty acids or bile acids at low pH through ionic bonds formed by its amino group [174]. In conclusion due to various reasons including the indigestible and viscous nature, the shield provided by oil droplets to prevent digestion from digestive enzymes such as lipase, chitosan has been regarded as a dietary fiber [170].

#### 4.10.3. Additive for Shelf Life Extension of Food

The food we consume is readily spoiled by the actions of microorganisms present in the environment and by oxidation when exposed to oxygen. Chitosan possesses the ability to scavenge free radicals present in food and thereby preserve the quality during storage. This ability depends on the deacetylation degree and the amount of unsubstituted amino groups [176]. However, chitosan with higher molecular weights displays lesser antioxidant activity due to the strong intramolecular hydrogen bonds present in its compact structure [177]. Antioxidants are substances that can slow down or inhibit oxidative degradation in food, which allows them to preserve food’s color and flavor while preventing the oxidation of vulnerable components. The current antioxidants used in the food industry are generally of low molecular weight and get degraded during processes such as heating and leaching [168]. On the contrary, antioxidant polymers such as chitosan are typically more resistant to this sort of deterioration or loss [170]. Chitosan’s capacity to chelate metal ions and attack free radicals is thought to be the foundation of its antioxidant properties. The presence of amino groups and hydroxyl groups in the chitosan structure, which gives this polymer a great propensity to donate hydrogen, is the cause of this action in both cases [168]. The chelating process encompasses the binding of metal ions with the hydroxyl group at the C6 and the amino group at the C2 of chitosan, thereby inhibiting the initiation of lipid peroxidation by metal ions. Similar reactions take place when free radicals interact with active hydrogen atoms in chitosan’s hydroxyl or amino groups to produce a very stable macromolecular radical, which hinders the progression of oxidative reactions [176]. A few more factors are regarded as affecting the antioxidant activity of a chitosan compound. Antioxidant activity decreases with increasing molecular weight and increases with increasing deacetylation degree [170]. Chitosan can be functionalized to increase its antioxidant activity without being hazardous or causing environmental harm by being coupled with flavonoids and phenolic acids utilizing oxidative enzymes [168]. The scavenging activities of water-soluble chitosan derivatives against the hydroxyl radical ·OH have been investigated by chemiluminescence technique and reported to exhibit IC50 values ranging from 246 to 498 mg/mL, which should be attributed to their diverse contents of hydroxyl and amino groups and various substituting groups [178].

#### 4.10.4. Chitosan as an Antimicrobial Agent

The degree of deacetylation, as well as the origin and level of polymerization, and environmental factors, particularly pH, all affect the antibacterial action of chitosan [169]. The amount of amino groups that are more such asly to interact favourably with the cell surface of microorganisms depends on the degree of deacetylation and pH [177]. Chitosan’s antibacterial activity is primarily caused by an electrostatic association between its polycationic structure and the primarily anionic components (lipopolysaccharides, peptidoglycan, and teichoic acid) of bacterial surfaces [168]. The primary mode of action of chitosan in Gram-positive bacteria is electrostatic contact with teichoic acids in the peptidoglycan layer, which causes disruption of various proteins and ultimately results in cell death. It has been proposed that chitosan tends to work in Gram-negative bacteria through two different mechanisms: chelation with divalent cations under an acidic pH reduces the stability of the membrane and nutrient uptake, and electrostatic interactions with the lipopolysaccharide at the outer membrane allow chitosan to transmit through the inner membrane, causing cell leakage [168]. As shown by Boi et al., grafting chitosan with groups that have antimicrobial effects, such as phenolic compounds, also enhances the polymer’s antimicrobial activity [168]. The mechanism of chitosan’s antifungal effect is comparable to that of its antibacterial action, and it appears to work against fungi that contain high amounts of polyunsaturated fatty acids [176].

Fresh or chopped fruits, berries, and vegetables that have been processed with chitosan solutions have a longer shelf life and are less such asly to develop mold and bacteria according to the research that have been conducted previously. Adding chitosan prevents bacteria from growing in cheese, hummus, and noodle dough as well [176]. Carrots [179,180], tomatoes [181], cucumbers [182], bell peppers [183], bananas [184], apples [185], pomegranates [186], and minced meat [187] all benefit from chitosan-based film coatings that effectively combat microbial spoilage to extend shelf life. A substantial synergistic antibacterial impact is seen for composite coatings based on the chitosan matrix that contain nanoparticles of inorganic antimicrobial agents such as silver [188,189,190] and zinc oxide [191,192].

#### 4.10.5. As an Emulsifier

Chitosan is a superior emulsifier for stabilizing heterogeneous oil-in-water systems. Chitosan tends to make the continuous phase more viscous, which renders it more difficult for dispersed particles to diffuse and slows the pace of droplet aggregation [176]. This characteristic is employed in developing of products such as sauces, desserts and ice creams. This characteristic is the result of chitosan’s amino groups being protonated in an acidic media, which gives the molecule an amphiphilic nature and enables adsorption at oil/water interfaces and the generation of emulsions by lowering interfacial tension [168]. The emulsifying ability of chitosan is strongly influenced by its molecular weight and deacetylation degree, and it is increased for low molecular weight chitosan with a deacetylation degree less than 60% or more than 86%; however, when the deacetylation degree is between 65% and 77%, its characteristics are greatly impacted by concentration. The digestion and stability of emulsified carotenoids can be improved by combining chitosan with soybean isolate protein. The fish oil emulsion in water is stabilized by a complex made of modified chitosan with lactoglobulin fibers. Additionally, stable pickering emulsions with corn oil are created when gelatin and chitosan interact electrostatically. Chitosan’s capacity to form micelles in aqueous solutions may be exploited for unstable substances as the sustainability of carotenoids and anthocyanins increases [176].

#### 4.10.6. As a Flocculant

Drinks made of solid suspended particles containing polyphenols, proteins, polysaccharides, and mineral components can be clarified using the characteristics of polycationite chitosan [193]. Since chitosan speeds up the sedimentation of suspended particles and improves the degree of bacterial and viral separation, it can be used to clarify fruit juices [194,195], fruit and grape wines, and beer [196], as well as at the preliminary drinking water purification stage [197].

#### 4.10.7. As a Functional Food Ingredient

Chitosan has been suggested as functional additives for food and feed products due to their documented positive health effects on both people and animals [170]. Health benefits of chitosan include reducing blood cholesterol and blood pressure, scavenging reactive oxygen species (ROS), protecting against infections, regulating arthritis, improving calcium uptake, and boosting anticancer capabilities. Additionally, due to its strong biocompatibility, emulsifying capacity, polycationicity, and hypoallergenic qualities, chitosan can be employed as a carrier for the encapsulation and controlled distribution of probiotic components in functional food items [176]. Chitosan may be utilized in the treatment of hypercholesterolemia and incorporated in the formulations of special meals with an anticholesterol effect since a number of clinical investigations have demonstrated that it binds to cholesterol [198,199].

Chitosan’s capacity to bind fat can be exploited in the development of dietary meals to slow down the rate of lipid digestion and absorption. Additionally, a composite film coating made of gelatin, chitosan, and gallic acid can be utilized to mimic fat in food products, replacing it by supplying organoleptic surface qualities [200]. Halder et al. investigated the hypocholesterolemic, antioxidant, and prebiotic effects of chitosan (60% deacetylated) in male albino rats. In comparison to animals that received the same amount of cholesterol-rich food but were not given such chitosan supplements, the levels of triglycerides, total cholesterol (TC), low-density lipoprotein cholesterol (LDL cholesterol), and atherogenic index (TC/high-density lipoprotein cholesterol) were significantly lower in animals fed on chitosan [168].

#### 4.10.8. Food Packaging

A critical environmental issue has been brought about by the widespread and unregulated usage of food packaging made of plastic with a petroleum base. The food packaging industry must therefore address this issue by developing novel, eco-friendly, and sustainable biodegradable polymers. An enormous amount of crustacean shell waste is produced annually as a major by-product of the seafood industry [201]. This waste can be used to produce value-added chitin, which can then be transformed into chitosan using a relatively straightforward deacetylation technique. After cellulose, chitin is the second most abundant biopolymer on earth and serves as the structural component of crustaceans, insects, and fungi [202]. Deacetylating chitin yields chitosan, a deacetylated derivative of chitin that can serve as a functionally versatile biopolymer due to the presence of amino groups, which are responsible for the polymer’s diverse biological and physical properties [202]. Chitosan is a desirable substitute for synthetic plastic polymers with a petroleum base given its antioxidant capacity, antibacterial activity, non-toxicity, biodegradability, and film-forming capabilities [203]. Chitosan-based biopolymers have been employed in developing active and intelligent packaging systems to preserve the quality of food [204]. Integrating nanoparticles, whiskers, and fibers, amalgamating the polymer with natural extracts, and using plasticizers and cross-linkers are some of the various strategies that have been incorporated into chitosan-based food packaging systems to improve food quality and safety, prevent the development of pathogenic microbes, and extend the shelf life of food [202]. The fundamental goal of such approaches is to enhance the mechanical, biological, and functional properties of chitosan-based food packaging. However, the industrial applications of this material, including food packaging, are severely constrained by its inherent flaws, which include low mechanical strengths, low thermal stability, and severe susceptibility to humidity [205].

#### 4.10.9. Chitosan and Blends with Other Biopolymers

A polymer blend is a compatible or phase-separated combination of at least two polymers or copolymers that is developed to improve the physical characteristics of each component [204]. Chitosan must be blended with other biopolymers, including polysaccharides, proteins, and lipids, in order to be applied as films and coatings and to increase its functionality as a food packaging material. Starch, cellulose, its derivatives, gums, proteins (from animals or plants), and lipids are some examples of these biopolymers [205]. Polysaccharide blends, in general, have a number of advantages over blends with proteins and/or lipids, including the low cost of the materials, the abundance of resources, and the relatively stable nature of the films.

Chitosan polymer blends can be mainly categorized into chitosan-natural biopolymer blends and chitosan-synthetic polymer blends [205]. By combining chitosan-based films with other natural biopolymers such as polysaccharides, proteins, and their derivatives, it is possible to enhance their functional qualities [206]. Pectin, starch (from rice, corn, potatoes, cassava, etc.), alginate, carrageenan, xanthan gum, xylan, glucose, kefiran, and its derivatives are examples of polysaccharides that can be coupled with chitosan, according to research. Due to their high abundance, acceptable mechanical properties, and excellent gas barrier properties to non-condensable gases (oxygen, carbon dioxide, and nitrogen) and aromas, protein-based films from plant and animal sources (soy protein isolate, corn zein, kidney bean protein isolate, quinoa protein, wheat gluten, etc.) have been studied for the development of biodegradable films [207].

The benefits of integrating chitosan with synthetic polymers (such as polyvinyl alcohol, polyvinyl pyrrolidone, polylactic acid, etc.) on the mechanical, biological, and physical properties of composite films have been thoroughly investigated in the food industry for packaging applications [205]. According to Bonilla et al. [208], the formation of intermolecular hydrogen bonds between the hydroxyl groups of synthetic polymers and the hydroxyl and amine groups of chitosan is the fundamental reason why some synthetic polymers and chitosan could be miscible. Polyethylene glycol, glycerol, silica, and ethyl vanillin, etc. are few of the compounds that are coupled with chitosan to form synthetic polymer blends in food packaging applications [205].

#### 4.10.10. Active and Intelligent Films Based on Chitosan

Chitosan is synthesized by deacetylation of chitin (poly (β-(1 → 4)-N-acetyl-D-glucosamine)), an important natural polysaccharide known since 1884 [204]. Chitosan is a semi-crystalline solid that are typically soluble in diluted organic acids, including acetic, citric, formic, lactic, malic, and tartaric acids [12,209]. One of the important chemical properties of chitosan is the degree of deacetylation (removal of an acetyl group). N-acetyl-glucosamine and D-glucosamine copolymers are formed when the acetyl (-C_2_H_3_O) group in the polymer chain is replaced by an amino (-NH_2_) group during the deacetylation process. Chitosan is the usual title for copolymers that contain more than 50% Dglucosamine units [203].

The primary causes of food product deterioration should be the key focus of novel food packaging solutions, which will also improve food safety. In order to have features and functionalities such as a specific gas barrier, gas or moisture absorbents, UV protection, antimicrobial capabilities, antioxidant activity, or monitoring capability to evaluate product quality, the packaging industry is aggressively searching for solutions. These new solutions are developed in such a way to increase food shelf life and enable longer-term product storage [210]. Active packaging and intelligent packaging are the two main cutting-edge packaging technologies that have developed to address this challenge [204]. An active film is one into which active components have been intentionally incorporated; these components release or absorb substances in order to prolong the shelf life of the food while preserving its quality and sensory attributes [211]. Active scavenging systems (absorbers) and active release systems are the two categories into which active packaging can be categorized (emitters) [204]. Active release mechanisms include CO_2_ emitters, antioxidant releasers, and antimicrobial packaging methods [211]. These active components, when combined with biodegradable polymeric matrices, have potent antibacterial and/or antioxidant characteristics and are suggested as a promising technique for developing food coatings or films [204]. For instance, chitosan films coupled with essential oils such as *Eucalyptus globulus*, bergamot, clove bud, or tree tea, among others, have demonstrated both antibacterial and antioxidant capabilities [212,213,214,215]. Moisture scavengers, oxygen scavengers, and ethylene absorbers are examples of active scavenging system methods [211]. Ethylene is a growth-stimulating hormone that can accelerate senescence and maturation by increasing the rate of respiration in food [216]. Scavenger’s properties can be imparted to chitosan films by incorporating compounds such as TiO_2_ nanoparticles [217]. Another approach is to integrate chitosan films with natural extracts such as gallic acid and compounds such as sodium carbonate to impart oxygen-scavenging properties [204].

Two main categories of chitosan-based intelligent films have been invented: (1) films with a visual color change brought on by colorimetric reactions, and (2) sophisticated biosensors. pH indicators, time-temperature indicators, and freshness indicators are all included in the first group. On the basis of the visible color shift that occurs along with the aggregation of gold nanoparticles due to their localized surface plasmon resonance, chitosan-gold nanoparticle composites can reveal the frozen condition and thermal history of food [218]. Materials that monitor pH alterations in food can detect microbial growth and oxidative deterioration as a result of physical-chemical changes to the food [219]. By adding an indicator of bromocresol blue and methyl red to a chitosan intelligent label, the CO_2_ during the respiration of fruits and vegetables, as well as microbes on their surface, has been monitored. A good linear relationship between CO_2_ concentration and the color change of the films at various pH values was found to be visible [220]. It is necessary to incorporate specific agents that are intended to improve certain biological and functional properties of active and intelligent chitosan-based materials. An improved polymer matrix compared with pure chitosan films may result from the inclusion of new components into the chitosan structure, which may also alter other physicochemical parameters such as mechanical strength, thermal stability, and water vapor permeability.

#### 4.10.11. Chitosan Film in Food Packaging

Chitosan is used in the packaging of food, either as flexible packaging films or coatings. Unlike coatings, which are often edible because they form a layer immediately on the top surface of the food, flexible films can either be edible or inedible [202]. Metallic ions, such as those of silver, zinc, copper, titanium, etc., are widely distributed in the natural environment. In some eukaryotes, including humans, the majority of these ions function as vital minerals. Additionally, compounds pose no threat to eukaryotic cells at concentrations below a threshold limit. In order to improve the mechanical, barrier, thermal, and photodegradation properties of the polymers used in food packaging, metal and metal oxide nanoparticles are used as fillers [202]. Additionally, these metal ions have shown remarkable antimicrobial and antioxidant properties in previous studies; hence, incorporation of these metals would impart additional benefits to the chitosan films. Commercial uses for silver salts as antibacterial substances have been extensively studied [221]. According to Priyadarshi et al. [222] silver ions cause the inactivation of respiratory enzymes, which results in the generation of reactive oxygen species and cell damage. Another investigation by Vimala et al. [223] revealed that silver nanoparticle-coated chitosan films had increased mechanical strength and antibacterial activity. Another prominent nanomaterial that is utilized increasingly in products for human consumption and is known to be safe is zinc oxide. Zinc oxide nanoparticles were synthesized and employed as fillers in the chitosan matrix by Priyadarshi et al. [222]. Due to the combination of chitosan and zinc oxide, the resultant films exhibit synergistic antibacterial action against *B. subtilis* and *E. coli*. Cárdenas et al. [224] compared the characteristics of copper nanoparticle-encrusted chitosan film to those of plain chitosan film and found that the latter had improved mechanical, barrier, and antibacterial activity.

#### 4.10.12. Flexible Packaging Films

The films’ characteristics are influenced by a number of variables, including deacetylation degree (DD), solvent pH, the type of acid employed, molecular weight, etc. For the films made with chitosan that had a lower DD value, a lower WVP was observed. Interestingly, the WVP dropped when the pH of the film-forming solution lowered. The water vapor permeability (WVP) levels are also considerably impacted by the type of acid utilized [202]. Flexible chitosan films are generally prepared by the solvent casting method. Chitosan is dissolved in appropriate solvents, typically in slightly alkaline water, and then poured onto a flat surface to allow the solvent to evaporate [225]. From the perspective of food packing films, the tensile qualities of the films are crucial. High-density polyethylene (HDPE), low-density polyethylene (LDPE), and cellophane can all be compared with the tensile strength (TS) and elongation at break (EB) values of chitosan films [226]. However, due to its high sensitivity to humidity and moisture, decreased thermoplasticity, decreased thermal stability, inability to be processed by industrial methods such as extrusion and molding, and inability to be stretched or heat sealed, chitosan’s use in food packaging is restricted [227,228]. Several attempts have been made to overcome these limitations, including combining the films with other natural or synthetic polymers or adding a variety of plasticizers, functional fillers, natural extracts, crosslinkers, etc. to the films.

#### 4.10.13. Films Infused with Natural Oils and Extracts

The search for biodegradable, biocompatible, non-toxic, and affordable biologically active compounds is expanding in significance as there is increased market competition to extend food shelf life while retaining environmental sustainability and economic viability. Natural essential oils and extracts are proven to be an excellent choice for this use because they may be made from agricultural waste or renewable plant components. Apart from imparting the films with antimicrobial and antioxidant activities, these essential oils can have plasticizing and water-resisting effects on the film, which is a major advantage [202]. Determining the antibacterial activity of essential oils requires consideration of their constituents, structures, and functional groups [229]. Thyme, cinnamon, rosemary, sage, basil, vanillin, oregano, and clove oils are the natural essential oils that consistently perform most effectively against foodborne pathogens [230]. In addition to having antimicrobial properties [231,232], these oils also have antifungal, antiparasitic, and antioxygenic properties [233,234]. In a different investigation, the chitosan-quinoa protein coating has also demonstrated better mechanical and water barrier capabilities [235]. Incorporation of mango leaf extract has also been proven to improve the mechanical as well as moisture barrier properties of food packaging films [236].

#### 4.10.14. Films Infused with Antimicrobial Agents

The development of packaging materials with biological activities, such as antibacterial, antioxygenic, and antioxidative, is being fueled by the growing research interest in advanced food packaging materials. According to Priyadarshi et al. [222] common antibiotics such as amikacin, clindamycin, vancomycin, and erythromycin were added to chitosan food packaging films to prevent the growth of *Listeria monocytogenes*, a common food pathogen, as well as other *Listeria* species. The effectiveness of the films was assessed, and a reduction in bacterial growth was observed due to the synergistic action of chitosan and antibiotics, particularly amikacin. According to Imran et al. [237] and Zimet et al. [238], chitosan-nisin blend films have antibacterial action, extending the shelf life of the packaged food product. The use of antibiotics in materials that contact food can result in major health risks, despite the fact that they are more effective for microbial inhibition. It is hypothesized that over time, the antibiotics may diffuse into packaged food products, resulting in indirect human consumption. As a result, human pathogens may evolve resistance to many drugs, and the human body may become extremely vulnerable to microbial diseases.

#### 4.10.15. Films Infused with Plasticizers and Cross-Linkers

A chemical known as a cross-linker aids in the formation of a cross-link between two polymer chains. Ionic or covalent crosslinking joins two polymer chains and modifies their characteristics, making them suitable for a variety of applications [202]. Although chitosan can be modified to have different properties using a variety of crosslinkers, including glutaraldehyde, formaldehyde, dialdehyde starch, citric acid, tannic acid, glyoxal, genipin, and quinone, only a few crosslinkers have been used in food packaging due to consumer health concerns. Jin et al. [239] developed genipin-crosslinked chitosan films, and their mechanical characteristics and solubility were investigated. The outcomes imply enhanced mechanical and elastic properties. In addition, the films were insoluble in both alkaline and acidic conditions. The primary target of developing these cross-linked polymers is to impart qualities including excellent dimensional stability, reduced creep rates, improved solvent resistance, increased glass transition temperature, etc. to food packaging films.

#### 4.10.16. Films Infused with Clays

Due to the remarkable ability of clays to disperse evenly in the polymer matrix, even at extremely minute concentrations, and to fill up the voids in the polymer matrix, giving rise to a very compact structure, polymer/clay composites have been gaining favor as a food packaging material. Due to the intercalation of clays, which forms a tortuous diffusion path for water vapor and gases, this compact structure exhibits remarkable barrier qualities. The reinforcing action of clays greatly enhances their mechanical properties as well [202]. Swain et al. [240] did a related study that emphasized the thermal stability and gas permeability of films made of Cloisite 15A that included chitosan. The nanocomposite films grew increasingly thermally stable and had less oxygen permeability as the concentration of clay in the films increased, making them a viable candidate for the application of food packaging [240].

#### 4.10.17. Films Infused with Polysaccharide Particles, Fibers, and Whiskers

A popular and efficient technique for improving the mechanical properties of polymeric films and sheets is the use of fillers for reinforcing. Chitosan, being a polymer with biodegradability, has gained popularity over other synthetic alternatives in the food industry. Yadav et al. [241] investigated how cellulose nanocrystals affected chitosan films and found that at 4% nanocrystal concentration, the mechanical and barrier properties had improved. Yang et al. [242] employed lignin nanoparticles as fillers in chitosan/PVA blend films for food packaging applications. Their research states that in addition to an improvement in mechanical and thermal stability, the films also acquired antimicrobial and antioxidant capabilities, making them a viable active food packaging material.

#### 4.10.18. Edible Coatings

The only films that are considered edible are those that are entirely made of food-grade materials, including the film-forming polymer matrix, the solvent in which it is dissolved, and any additives such as fillers, plasticizers, crosslinkers, or biologically active agents that are incorporated into the films [243]. Edible coatings can be applied to food surfaces directly using a variety of techniques, such as dipping, spraying, and panning, or by combining food and coating solution in a rotating container, followed by drying [202]. In addition to being used singly, chitosan is frequently combined with other biopolymers or additives in edible coatings. Research on chitosan-based edible coatings for unprocessed or lightly processed food has been presented in numerous studies. In a study on strawberries, they were coated with chitosan and oleic acid in cold storage, and it was shown that the coatings preserved the mechanical characteristics and color of the fruit [244]. The impact of chitosan coatings embedded with Ag-chitosan composite nanostructures on the quality of freshly cut melons was investigated by Ortiz-Duarte et al. [245]. It was found that the coating treatments decreased the fruit’s respiration rates, prolonging the fruit’s shelf life. The versatile films or coatings based on various formulas and their uses are tabulated in Table 4.

### 4.11. Wastewater Purification

#### 4.11.1. Removal of Heavy Metal Ions

**Figure 8:**
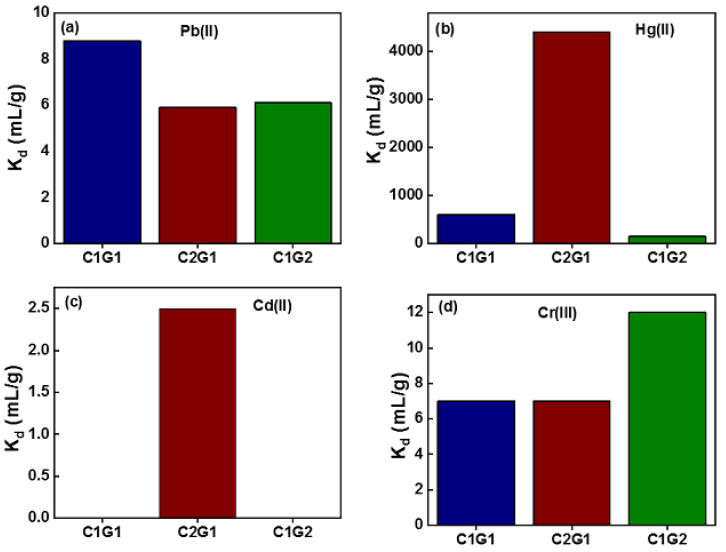
K_d_ values for oven-dried hydrogel particles C1G1, C2G1, and C1G2 in single metal ion solutions of (**a**) Pb(II), (**b**) Hg(II), (**c**) Cd(II), and (**d**) Cr(III). Figure adapted from Ref. [261].

Perumal et al. [261]., reported the removal of multiple heavy metal ions from water using chitosan/gelatin hydrogel particles that were synthesized by mixing varying ratios of chitosan to gelatin in a span 85 surfactant solution as 1:1 (C1G1), 2:1 (C2G1), and 1:2 (C1G2), followed by removal of the surfactant by washing with hexane. The obtained hydrogel particles were dried in two ways: oven drying at 60 °C and freeze drying. Oven-dried hydrogel particles were tested for the adsorption of Pb (II), Hg(II), Cd (II), and Cr (II) (Figure 8). The non-equilibrium distribution coefficient value (K_d_) for the adsorption of Hg(II) ranges 136–4450 mg/L by chiton/gelatin hydrogel synthesized in all proportions, while the K_d_ for the adsorption of Cd(II) is nearly zero and that of Pb(II) and Cr(II) is very low, suggesting that the hydrogel particles adsorb Hg more efficiently. This observation has been further explained by concentrating on the binding energy of the Hg-Ligand bond where the ligand consists of N, S, and O atoms. Hg-N binding energy is higher than that of Hg-S and Hg-O, and hence the hydrogel with the highest chitosan content, where the hydrogel is enriched with more nitrogen, has shown to remove more Hg(II). All the hydrogel compositions were effective in removing all metal ions in higher concentrations (54–95%), which is different from the behaviour observed in removing metal ions when they are present individually, where only Hg was removed highly (>50%) while the removal of other metal ions was very low (<10%). However, the removal of Hg from the metal ion mixture was more prominent than that of the other metal ions, being similar to the trend observed with the removal of metal ions when present individually [261].

**Figure 9:**
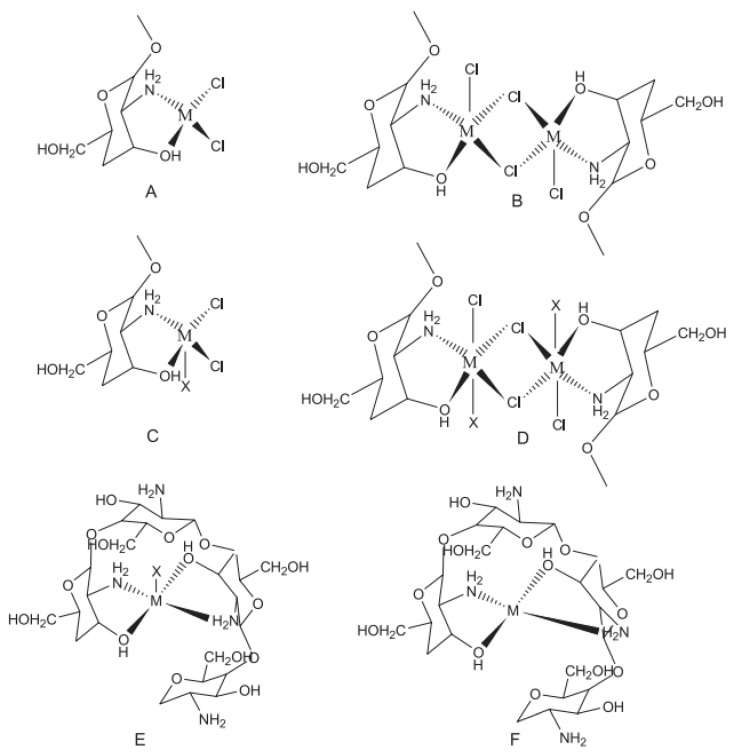
Chelation of metal ions by chitosan in different geometries. Figure adapted from Ref. [265]. (**A**) tetragonal (**B**) and (**C**) pentagonal (**D**) hexagonal (**E**) pentagonal (**F**) tetragonal geometries.

Karimi et al. [262]., studied the effect of EDTA-functionalized magnetic chitosan/Al_2_O_3_/Fe_3_O_4_ on the removal of Cu, Cd, and Zn, where functionalization with EDTA has increased the adsorption capacity by 9.1, 5.6, and 14.3 times, respectively. The maximum adsorption capacity was found for the adsorption of Cd, followed by Cu and Zn, where 99.98, 93.69, and 83.81% of the above metal ions, respectively, have been removed. Metal ion adsorption has followed pseudo-second-order kinetics, suggesting chemisorption of the metal ions to the polymer, and the adsorption of all the metals followed the Langmuir model further, indicating that monolayers of metal ions have been adsorbed to the EDTA functionalized magnetic chitosan/Al_2_O_3_/Fe_3_O_4_ [262]. Furthermore, chitosan incorporated composites have also shown to be effective in adsorbing metal ions. Chitosan/sodium alginate/calcium ion double-network hydrogel has shown to be effective in removing metal ions including Pb^2+^, Cu^2+^, and Cd^2+^ with adsorption capacities of 176.50 mg/g, 70.83 mg/g and 81.25 mg/g, respectively [263]. Diethylenetriaminepentaacetic acid-modified chitosan/polyethylene oxide nanofibers effectively adsorbed Cu^2+^, Cd^2+^ and Ni^2+^ in the order of Cu^2+^ > Pb^2+^ > Ni^2+^ with adsorption capacities of 177 mg/g, 142 mg/g, and 56 mg/g, respectively, following pseudo-first-order kinetics and metal ions adsorbed s monolayers following the Langmuir isotherm model [264]. Chelation of metal ions by chitosan in different geometries are shown in Figure 9.

#### 4.11.2. Removal of Dyes

**Figure 10:**
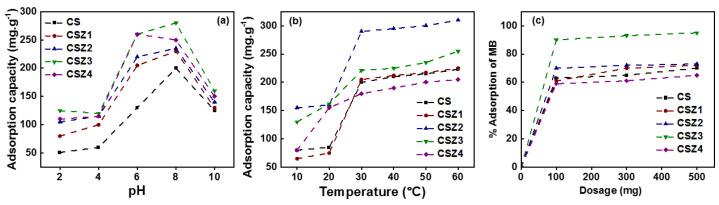
(**a**) Effect of pH (**b**) Temperature (**c**) adsorbent dosage on the adsorption of MO to chitosan/sodium zeolite adsorbents. Figure adapted from ref. [266].

Dyes released from industries including textile, paint, paper, food, cosmetics, etc. cause harsh environmental impacts, so their elimination is of great importance. Chitosan-containing products have been introduced recently to remove dyes from water. Ragab et al., studied the removal of Brilliant green dye using nano hydroxyapatite/chitosan where the highest percentage adsorption of the dye (99.5%) was obtained at neutral pH 7 and at highly acidic medium the adsorption was very low due to the dissolution of the adsorbent in acidic medium and the percentage adsorption was high at neutral and alkaline pH. Adsorption of the dye increased with increasing contact time and adsorbent dosage. The data were well fitted to the Dubinin–Radushkevich isotherm model and the first order kinetics model, and increasing dye adsorption with increasing temperature reveals that the dye adsorption is endothermic [264]. Hussain et al. [266]., reported the sequestration of methyl orange by chitosan-coated sodium zeolite composite films prepared by varying the weight of sodium zeolite incorporated as 1, 2, 3, and 4 g per 1 g of chitosan, and they were denoted as CSZ1, CSZ2, CSZ3, and CSZ4, respectively. Composite film CSZ3 showed the highest adsorption capacity of 285 mg/g at pH 8 (Figure 10a). The adsorption was lower in acidic pH due to the dissolution of chitosan and protonation of MO in acidic medium, which cause repulsions with the positively charged chitosan-zeolite films. Adsorption drastically decreased at pH 10 due to the high concentration of OH- present which competed with the MO molecules for the surface of the adsorbent, and the production of amino groups.

As a result of the deprotonation of NH_2_ groups, which produce repulsive forces between the negatively charged adsorbent and MO. The adsorption capacity increased from 157 mg/g to 302 mg/g when the temperature was increased from 10 to 60 °C in 10 °C intervals, and a drastic increase was observed moving from 20 to 30 °C where the adsorption capacity increased from 161 to 287 mg/g, and with further increase in the temperature, the adsorption capacity slightly increased (Figure 10b). The rise in the degree of hotness increases the mobility of MO molecules in the blend and reduces the thickness of the blend, which thereby amplifies the adsorption of MO molecules to the exterior layer of the film and further facilitates the migration of MO molecules into the porous structure of the films. Moreover, an increase in temperature facilitates the interactions between the adsorbate and the functional groups of the adsorbate, leading to higher adsorption capacities. The adsorption capacity increased with increasing adsorbent dosage (Figure 10c), where a significant increase was obtained when the dosage increased from 1 g/L to 2 g/L due to the increased availability of the active sites, and with further increases, the adsorption capacity increased slightly due to the increased covering of the functional sites of the adsorbent due to the high crowding of the adsorbent particles. Adsorption of MO to chitosan/sodium zeolite followed pseudo-second-order kinetics with a rate constant of 0.000125 min^−1.^ Furthermore, the adsorption followed the Freundlich model, revealing the multilayer formation of MO on the adsorbent. Hence, synthesized chitosan/sodium zeolite composites were effective in removing MO from water [266].

More chitosan-based composites have removed dyes from wastewater. Malachite green has been removed by SDS/coir pith activated carbon/chitosan [267], EDTA-functionalized magnetic chitosan has been effective in removing both methylene blue and Pb(II) and Cu(II) [268], a magnetic graphene oxide/chitosan composite has been used to remove both reactive blue 19 and Ni(II) [269], and a polyurethane/chitosan composite was effective in removing food dye 17 [270].

#### 4.11.3. Removal of Pesticides

The level of pesticides being used has been drastically increased due to the rapid development of the agriculture industry. The need for removing them is more emphasized as they are hazardous to human beings. Chitosan derivatives have been widely applied to remove pesticides from contaminated water. Mostafa et al. [271] reported the use of chitosan/zeolite composite to remove acephate, omthosate, and methyl parathion, which are three species of organophosphorus pesticides with adsorption capacities of 650.7 mg/g, 506.5 mg/g, and 560.8 mg/g, respectively, following pseudo-first-order kinetics, and the pesticides adsorb to the adsorbate as monolayers as confirmed by the data best fitted to the Langmuir isotherm model. Adsorption was spontaneous, feasible, and exothermic, and the composite showed promising activity in adsorbing the pesticides in the presence of inorganic anions such as SO_4_^2−^, PO_4_^3−^, and NO^3−^, as well as the metal ions Zn^2+^, Pb^2+^, and Cd^2+^. Though the effect of the existence of SO_4_^2−^ was insignificant, other ions showed competitive behaviour where the percentage adsorption decreased considerably. Chitosan/Zeolite composite has shown to be stable in removing the organophosphorus pesticides for five runs [271]. A magnetic chitosan/activated carbon bio-nanocomposite with a UiO-66 metal organic framework has shown to be effective in adsorbing Imidacloprid pesticide and Co^2+^ and malachite green dye. The adsorption capacities of the above three pollutants in a ternary medium are 25.2 mg/g, 44.5 mg/g, and 62.1 mg/g, respectively. Adsorption followed pseudo-second-order kinetics, indicating the chemisorption of adsorbates, and the adsorbates adsorbed as monolayers, as suggested by the data well fitted to the Langmuir isotherm model. The fabricated composite is an eco-friendly, cost-effective, reusable adsorbent that can be potentially used to remove multiple pollutants simultaneously [272]. Chitosan has been used to remove pesticides in grape juice, where 98% of chlorpyrifos and 97% of ethion have been removed by a 0.5% chitosan solution in 1 h and 96%, 95%, 94%, and 86% of phorate, fenthion, fenitrothion, and diazinon, respectively, were removed by a 1% chitosan solution in 2 h [273]. Ghimici et al., reported the pesticide removal efficiency of thymine-containing chitosan derivatives by flocculation/coagulation. Fastac 10 EC and Karate Zeon, of which alpha-Cypermethrin and lambda- Cyhalothrin, respectively, are the active pyrethroid ingredients; Novadim Progress, where Dimethoate is the active organophosphoric ingredient; and the Bordeaux mixture have been selected as the pesticides. Chitosan-thymine coordination was effective in removing 80–85% of Karate Zeon, 90% of Fastac 10EC, and Novadim Progress, and 95% of Bordeaux mixture. Fastac 10 EC and Karate Zeon were removed by flocculation due to the charge neutralization, and Novadim Progress and Bordeaux mixture was removed by flocculation through the synergetic effect of charge neutralization and hydrogen bonding or copper ion binding to the amine and thymine groups [274].

#### 4.11.4. Removal of Pharmaceuticals

Pharmaceuticals that entered the water reservoirs by various means should be eliminated as they cause advanced toxic effects to living beings as they accumulate in the ecosystem. Chitosan-based purifying systems have been developed recently to remove pharmaceuticals from water. Tzereme et al., studied the effect of carboxyl grafted chitosan on the removal of diclofenac. Succinic anhydride, maleic anhydride, itaconic anhydride, and trans-aconitic acid were used as the grafting agents, and the resulting carboxyl-grafted chitosan removed 80.9%, 66.2%, 89.5%, and 92.8% of diclofenac, respectively [275]. Graphene oxide-chitosan and amine-graphene oxide-chitosan hydrogels were also shown to be effective in removing 90.42% and 97.06% of diclofenac, respectively [276]. Chan et al., learned that DNA-chitosan hydrogel was capable of removing Paracetamol, Phenazone, 3,3′-Dihydroxydiphenyl amine, Clofibric, Carbamazepine, Thymol, Ibuprofen, and Ketoprofen pharmaceuticals from water; additionally, the hydrogel was effective at adsorbing dyes such as methylene blue and congo red, and metal ions, including Hg^2+^, Pb^2+^, Cd^2+^ and Cu^2+^ [277]. Cross lined magnetic chitosan/Activated biochar effectively removed 96.4% of diclofenac, 98.8% of Ibuprofen and 95.2% of naproxen [278].

### 4.12. Controlled Release Fertilizers

**Figure 11:**
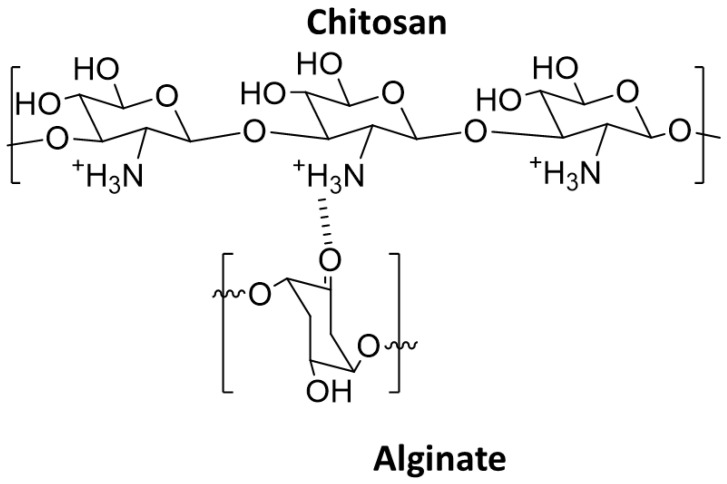
Chitosan-alginate complex.

Urea, the most promising nitrogen-releasing fertilizer, is widely used worldwide but causes environmental pollution due to its excessive use. Furthermore, loss of urea in the agricultural fields due to rapid evaporation has become a trending problem for the growers. Therefore, the slow release of urea is an essential concept being researched to mitigate the problems above mentioned. Vo et al., reported a new fertilizer formula designed to slow-release urea using a hydrogel fabricated by cross-linking chitosan and poly (vinyl alcohol) (PVA). Formulations were made in different PVA and Urea compositions, and water absorbency and release of NH_4_^+^ were measured. Water absorbency increased drastically upon immersion in water, which gradually decreased due to the dissolution of urea and polymer molecules that were not cross-linked and the hydrolysis of the hydrogel. Low degree of cross-linking resulted in a lower content of glutaldehyde, which causes higher water absorbency because more water molecules are entered into the more spacious structure with less cross-links. The higher degree of cross-linking resulted in high hydrolytic stability but low release of urea, whereas a sustained release of urea from the formulations was observed for a prolonged period of 10 days. The release of urea from low-urea content formulations followed the first-order and Hixson–Crowell models and the release of urea from high urea included formulations followed the Higuchi and Korsmeyer–Peppas model [279]. Furthermore, encapsulation of urea in chitosan helped to gain a potato yield of 75 Kg N ha^−1^ by applying 25% of the required level of N fertilizer, and this fertilizer formula reduced viable counts of soil bacteria involved in nitrogen transformation. The soil nitrogen content, both ammonical and nitrate, was lower than that of the conventional urea treatment. Moreover, nitrogen-cycling microorganisms were greatly reduced due to the efficient controlled release of urea from the encapsulated chitosan polymer [280]. Similarly, slow release of urea by chitosan-alginate composite is shown in Figure 11. Beads that followed a quasi-fickian diffusion mechanism were reported by Sathisaran et al. [281]. The Montmorillonite-Chitosan nanocomposite increased the release of phosphorous and potassium from 22.0 to 94.9% and 9.6 to 31.4%, respectively, and 55.3% of the total incorporated fertilizers were released over 15 days compared with the traditional fertilizer powder, which released 89.2% of the fertilizer during the first irrigation [282]. Kubavat et al., studied the slow release of potassium by chitosan nanoparticles and found that this fertilizer formulation improves some properties of soil, including enhanced porosity, higher water conductivity, increased friability that facilitates root growth and decreases the dry density and thus improve the nutrient uptake of plants [283]. Slow release of potassium from chitosan/ montmorillonite microparticles has been observed by Messa et al., where a 52% decrease in the swelling ability resulted which reduced the nutrient release [284]. Hence, it is evident that nutrients, including N, K, P are sustainably released once they are loaded to chitosan and chitosan derived polymers.

### 4.13. Dye Sensitised Solar Cells

The main demerit associated with dye-sensitised solar cells is the leakage and vaporization of liquid electrolyte, which restricts long-term usage of the cells. To overcome such a challenge, Chawla et al., have assembled a cell using an electrolyte system comprised of chitosan as the host polymer. The solar cell conversion efficiency obtained was about 1.8% with a fill factor of 53% using an irradiation of 100 mW/cm^2^ at 25 °C. The cell was stable for 10 weeks with no leakage or vaporization, compared with the same cell with liquid electrolyte, which was stable for only 6 weeks. Thus, the chitosan polymer electrolyte has improved the stability of the cell, and moreover, as chitosan is a natural polymer, the fabricated cell is ecofriendly [285]. Rahman et al., reported the use of chitosan biopolymer laden with NaI salt as a solid polymer electrolyte for dye-sensitised solar cells. Semi crystalline domains of chitosan are weakened by the interactions between chitosan and NaI and thus favor the conduction of the redox shuttle ions between the electrodes of the cell. Solid-state polymer electrolyte-baring dye-sensitised solar cells were stable even after a month with no loss in activity, but the liquid-state electrolyte-based cells 85% of their initial performance [286]. Similarly, chitosan incorporated dye-sensitised solar cells have been fabricated and have used efficiently by Areerob et al., who reported the fabrication of a counter electrode by gamma irradiated chitosan-doped reduced graphene-CuInS_2_ composite [287], Zulkifli et al., who fabricated a dye-sensitised solar cell assembled from modified chitosan-based gel polymer electrolytes incorporating potassium iodide [288], and Praveen et al., who created a ZnO/ZnS nanocomposite based dye-sensitised solar cell with chitosan polymer electrolyte [289]. As reported by Zheng et al., chitin-derived nitrogen-doped porous carbons with a high specific area and a hierarchically porous nature have shown to be effective as high-performance super capacitors [290]. Similarly, Wei et al., also reported the use of chitosan in a sandwich-like chitosan carbon spheres/MXene composite for supercapacitors [291].

### 4.14. Water Availability in Plants

Water availability is a significant regulator of plant growth that triggers the hydropattern process, which promotes root branching. Lateral branches of a plant are directed towards regions of high-water availability, hindering the growth in less water-available area. The mechanism of hydropatterning that determines the special perception of plants is yet to be fully explored. A number of stressors that affect the growth of a plant are discussed under ‘Prevention of the abiotic stress in flora’. In this chapter, the role of chitosan in regulating water availability will be discussed specifically [292]. Water is essential for the development of plants. In contrast to nutrients, which are taken up by roots and leaves and incorporated into the dry mass of leaves via chemical reactions, the water absorbed by plant roots is lost to the atmosphere through transpiration in plants. About 0.1–1% the absorbed water remains in the plant in the form of water or other chemical substances [293].

According to reports, water productivity, or the amount of water required to produce a unit mass of crop dry matter, ranges between 1 and 10 m^3^ per kilogram of grain production [294]. Several studies have been conducted to evaluate the potential of chitosan as a prospective treatment to increase water availability in plants. It has been found that water stress can retard the growth of cowpea plants and reduce the ion percentage, carbohydrate content, and chlorophyll content in the shoot. Water stress can affect the yield of the crop as well as the quality of the crop. In comparison to untreated plants, foliar-applied chitosan, 250 mg/L, enhanced plant growth, yield, and quality as well as physiological parameters in plant shoots under stressed or non-stressed conditions. Anatomically, water stress reduced the thickness of the midrib vascular bundle, the mesophyll tissue and the midrib region of the leaf blade. In particular, 250 mg/L of chitosan treatment and its interactions with stress conditions enhanced all of the aforementioned parameters in either stressed or non-stressed plants. Application of 250 mg/L chitosan is said to have lessened the severity of water stress-related damage to cowpea plants [295].

The results of another study proved that water stress hindered the growth of common bean (*Phaseolus vulgaris* L.) plants, lowered the concentration of carbohydrates in the shoots, and reduced the content of nutrients and photosynthetic pigments. Additionally, water stress has an impact on the crop’s productivity and quality, as shown by the amounts of nutrients, proteins, and carbohydrates present in the plant. In comparison to chitosan-untreated plants, foliar-applied chitosan, 200 mg/L, boosted plant growth, yield, and quality as well as physiological components in plant shoots under stressed or non-stressed conditions. Hence chitosan can be regarded as a potential substance that could be utilized to lessen the adverse effects of water stress on the growth and yield of *Phaseolus vulgaris* plants [296].

Another study was carried out to evaluate the effects of S-nitrosoglutathione (GSNO)—a nitrogen oxide donor—supplying to sugarcane plants under water deficit. In comparison to providing GSNO in its free form, it was predicted that GSNO encapsulated into nanoparticles would be more efficient at decreasing the impacts of water deficits on sugarcane plants. Additionally, chitosan nanoparticles containing GSNO were created and characterized and the effect on water deficit was reported. The following treatments were applied to sugarcane plants after they had been cultured in nutrient solution: control (well-hydrated); water deficit (WD); and WD + GSNO sprayed in its free form (WDG) or encapsulated (WDG-NP). Both GSNO formulations generally mitigated the impact of a water shortage on sugarcane plants. In contrast to plants that received free GSNO, the encapsulation of this donor into chitosan nanoparticles led to increased photosynthetic rates under water stress [297].

Another study was conducted to explain the viability of using chitosan to induce tolerance to water deficit in several maize (*Zea mays* L.) hybrids, while contrasting tolerance to water restriction, tolerance, and sensitivity. At the pre-flowering growth stage, the maize plants were stressed for 15 days with water deprivation and foliar applications of various chitosan concentrations (60, 100, 140, and 180 mg L^−1^). Biophysical parameters, such as water potential, relative water content, chlorophyll content, gas exchange, and biochemical assays, were quantified based on the activities of SOD, CAT, APX, and PAL antioxidant enzymes, lipid peroxidation activity, and hydrogen peroxide content in order to understand the induction behavior of the tolerance to water restriction. Among the treatments, maize plants exposed to 140 mg L^−1^ of chitosan foliar application exhibited similar behavioral responses to plants growing in a favorable watering condition. These favorable results are attributed to the high level of antioxidant enzyme activity and gas exchange and the low amounts of hydrogen peroxide and lipid peroxidation [298].

The mode of action of chitosan in increasing the water availability of plants is not fully elucidated yet. Due to the homogeneity of positive charges along its length, this biopolymer can have an enormous variety of potential action paths, enabling a wide range of interactions with the other molecules in the cell matrix [298]. Possibly as a result of the several metabolites that chitosan is capable of producing, which lower transpiration and increase the amount of water accessible to plants for enhanced growth and production [296]. The fact that chitosan, when deposited in the cell wall, encourages a reduction in stomatal conductance, increasing the leaf’s resistance to water vapor loss, and ultimately improving the water use in plants to assimilate carbon and the production of biomass, may explain the role of chitosan in improving the water availability of plants. Another method of reducing water loss through perspiration is to use reflective antiperspirant action on the leaves, which increases solar reflection and cools the leaves through evaporative cooling [299].

### 4.15. Preventing of the Abiotic Stress in Flora

Due to the changing climate, urbanization, shrinking amount of arable land, depletion of natural resources, constantly increasing global population, etc., the agriculture industry is currently facing a number of challenges. Therefore, it is essential to increase agricultural productivity by utilizing cutting-edge and environmentally friendly approaches. In the recent past, chitosan and chitosan-coupled materials have emerged in the field of agriculture due to their biocompatibility, less-toxicity, bioactivity, abundance in nature, and antimicrobial properties. Given its significant ability to regulate various elements of plant physiological function, it has been widely utilized as a bio-stimulant and an elicitor. Chitosan combined with nanotechnology has proven to exhibit remarkable results in improving plant growth and development. Moreover, through the regulation of defense-related genes and the elevation of secondary metabolites, chitosan nanoparticles (NPs) have been shown to trigger innate immune responses to fight abiotic stress in plants. Chitosan also has the ability to induce an antioxidant defense mechanism to alleviate the toxicity that is caused by abiotic stress in plants. The use of chitosan in agriculture has been researched in a variety of crop species, including cereal, medicinal, fruit, and ornamental crops. Chitosan can be considered a desirable contender for agricultural applications because of its intrinsic cationic polymer properties, which include bio-adhesion, cellular transfection, anti-inflammatory, and anti-hypercholesterolemic properties [300]. Depending on the species of plant, the concentration of chitosan molecules, and their stage of development, it impacts various physiological responses in plants. Effective outcomes against diverse abiotic challenges found with chitosan-treated plants are shown in Table 5.

### 4.16. Controlling Salinity Stress

Salinity is a significant abiotic stress factor that has a global effect on plant growth in the field of agriculture and has an impact on the entire plant, both physiologically and biochemically. In extreme cases, it can also prevent plants from absorbing nutrients and water, either through low external osmotic potential or from the toxic effects of a higher accumulation of Na^+^ and Cl^−^ ions as a result of direct ionic effects. Salt stress alters biochemistry and produces reactive oxygen species (ROS), which interfere with cellular function and cause oxidative stress. Numerous studies have discovered that increased malondialdehyde (MDA) accumulation was observed in salt-affected plants, which is mostly due to membrane lipid peroxidation brought on by ion toxicity [315]. Malondialdehyde is typically used to measure membrane lipid peroxidation in plants. MDA is a frequently used indicator of oxidative lipid damage brought on by stress from the environment [316]. The detrimental effects brought on by salt stress, however, may be mitigated by treatment with chitosan at low concentrations, according to many researchers. 0.0625% oligochitosan treated seed had positive effects in a hydroponic experiment on wheat by significantly increasing antioxidant enzyme (catalase (CAT), peroxidase (POD), superoxide dismutase (SOD)) during salt induced stress and being able to reduce oxidative stress. One of the beneficial oligosaccharides is oligochitosan, which is made of b-1, 4-linked 2-amino-D-glucose units and contains trace amounts of 2-acetomido-D-glucose production units by chitosan degradation [311]. Comparably, pretreatment with chitosan during salinity stress results in increased antioxidant enzyme activities and a lower level of MDA content, which ultimately reduces the negative effect caused by salt stress in *Oryza sativa*, *Zea mays* [310]. Vigna radiata [310], Trachyspermum ammi [307]. By lowering enzyme activity in both crops, seeds of safflower (*Carthamus tinctorius* L.) and sunflower (*Helianthus annuus* L.) treated with low concentrations of chitosan are able to reduce the oxidative stress brought on by salt stress [307].

Due to its high surface-to-volume ratio, better penetrability owing to the nano size, and ability for more interactions with biomolecules and complexes within cells, nanochitosan may have a more profound impact on plants compared with other bio-stimulants available.

In order to prevent salt stress in maize, nitric oxide (NO) releasing chitosan nanoparticles were found to be superior to free NO donors [317]. Nanochitosan-infused solid matrix priming of mung bean seedlings has been demonstrated to reduce the adverse effects of salinity stress and enhance the plants’ growth, metabolism, protein levels, and chlorophyll content [318]. When exposed to 0.1%, 0.2%, and 0.3% nanochitosan at a salt concentration of 100 mM, the salt-sensitive bean plant *Phaseolus vulgaris* L. demonstrated increased seed germination [319]. When applied to tomato plants under salt stress, chitosan-polyvinyl alcohol hydrogels with and without copper nanoparticles increased the expression of genes for the synthesis of jasmonic acid (JA) and superoxide dismutase (SOD), which are essential for detoxification and plant growth [320]. To fully unveil the potential of nanobiotechnology, and to maximize the use of chitosan as a nanobiostimulant to counteract abiotic stress in a variety of plant species under a variety of agrochemical settings, further study needs to be conducted.

### 4.17. Controlling Heat and Drought Stress

Abiotic stressors such as dryness and temperature stress can be mitigated by chitosan. Since it commonly occurs simultaneously with drought stress and is challenging to monitor, heat stress is frequently viewed as a complex issue [321]. Lower yields might result from a plant’s water deficiency, which can also impair its morphology, physiology, and biochemistry [322]. Drought stress can induce the closure of stomata in plant cells, thereby leading to the reduction of CO_2_ intake, which has a direct impact on plant growth and photosynthesis [323]. Drought stress can also destroy the chloroplasts and reduce the chlorophyll content of crops. It also reduces the activity of the enzymes in the Calvin cycle in photosynthesis [323]. When grapevine stem cuttings were dipped in 0.5% or 1.0% (*w/v*) chitosan, they developed resistance to low and high temperatures, respectively, while the 1.0% (*w/v*) chitosan treatment developed resistance to drought by the maintenance of chlorophyll content under drought stress [301]. Chitosan seed priming improved the germination index and time at low temperatures and achieved significant seedling growth in treated seeds compared with untreated seeds, which boosted the chill tolerance of maize [324].

When applied to the leaves of pearl millet under drought stress, nanochitosan emulsion has been found to improve plant water status by reducing stomatal conductance and transpiration [325]. Foliar application of chitosan to wheat with water stress has shown to lower the harmful effects on plant yield and plant growth [322]. N-succinyl chitosan and O-dicarboxymethylated chitosan derivatives have shown to improve the water stress tolerance in hybrid maize crops that is typically sensitive to drought stress.

### 4.18. Controlling Heavy Metal Toxicity

Another variable that has an impact on agricultural land use is metal-contaminated soil. According to numerous studies conducted previously, it has been proven that chitosan can form complexes with non-nutrient elemental ions, including a variety of heavy metals, due to the presence of functional amino and hydroxyl groups. There have been studies on the use of bulk chitosan to alleviate the stress heavy metals exert on the growth and development of plants. According to Kamari et al. [326,327] the use of chitosan led to the binding of Ag, Zn, Cd, and Pb and the accumulation of metals in perennial rye grass and rapeseed. The effects of chitosan and chitosan oligosaccharides in phytoremediation and biofortification strategies were thoroughly examined by Vasconcelos [328]. These findings offer a potential mechanism for reducing phytotoxicity in the presence of heavy metals, and it can be speculated that while chitosan and its oligomers can absorb more toxic substances, they may also boost the absorption of vital minerals. It has also recently been shown that foliar application of different molecular weight chitosan, such as 10 kDa, 5 kDa, and 1 kDa, can reduce the harmful effects of cadmium in a hydroponically cultivated edible rape, *Brassica rapa* L. [329]. Chitosan and its derivatives have been shown in a study by Kamari et al. [326] to efficiently bind metal ions (Ag(I), Pb(II), and Cu(II)) in soil that co-exist with other ionic substances, including K^+^, Cl^−^, and NO_3_^−^. However, there is a scarcity of research on the use of nanochitosan to reduce the stress brought on by heavy metal toxicity.

## 5. Toxicity of Chitosan

Chitosan is a biodegradable polymer whose biodegradability could occur through chemical or enzyme catalysis. The degradability depends on the degree of deacetylation and the availability of amino groups. The toxicity of chitosan depends on the charge density and degree of deacetylation. Kazemi et al. evaluated the cytotoxicity of the thiolated chitosan-lauric acid as a new chitosan derivative by MTT assay. The cytotoxicity of the new polymer and the normal chitosan were not significantly different, as indicated by the cell viability studies performed against normal gingiva human cells (HGF1-PI 1). Chitosan derivative, thiolated chitosan-lauric acid, shows low toxicity due to the intramolecular H bonds, which lead to low flexibility where the rigid structure limits the interactions between the cell membrane and the positive charge of the polymer, rendering it less toxic. On the other hand, lauric acid conjugation to chitosan increases its viability and biocompatibility [330]. Chitosan nanogels were found to be free of cytotoxicity when tested against the HEK-293 normal cell line by MTT assay [331]. Asghar et al. evaluated the cytotoxicity of green synthesized chitosan coated silver nanoparticles using the HeLa cell line using the MTT assay method and found that 93.2% cell viability was achieved with 100 µg/mL but with higher doses (300 and 400 µg/mL), the viability reduced to 54.5% and 35.2%, respectively. However, the chitosan-coated Ag nanoparticles showed less toxicity compared with Ag nanoparticles, and they exhibit more antibacterial, anticoagulant, antiplatelet, and thrombolytic activities compared with Ag nanoparticles [332]. Furthermore, Frigaard et al. reported that among the 25 articles they have referred to except one, all others reported less toxicity of chitosan nanoparticles with >80% cell viability when studied both in vitro and in vivo [333], and the only study where low cell viability was reported was the use of dry powder of chitosan nanoparticles (250 and 500 µg/mL) with Calu-6 cells for 24 h [334]. Therefore, it is evident that chitosan and its derivatives are less toxic to human cells.

**Scheme 3:**
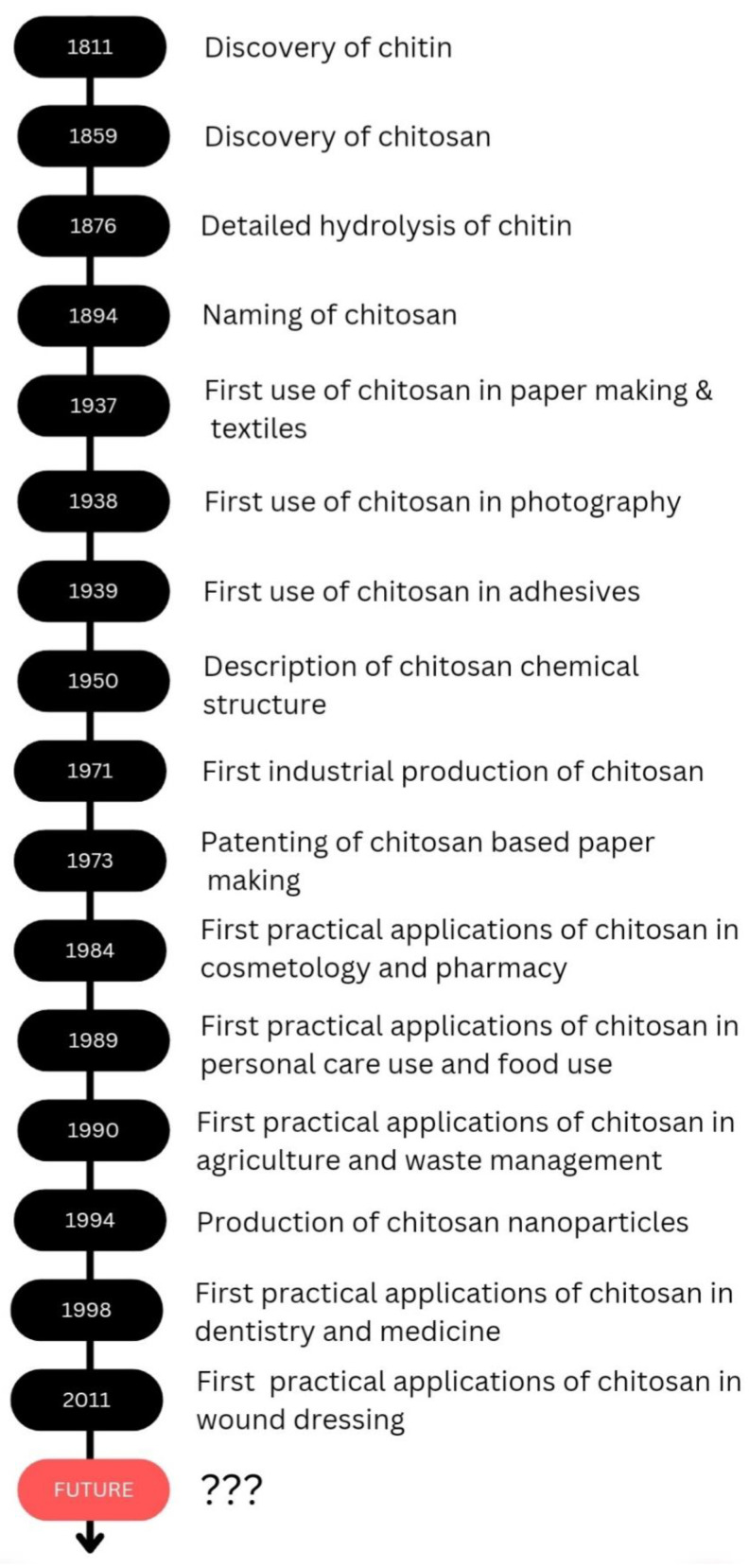
The discovery and the progress of chitosan and its applications.

As revealed by the examples above throughout the review, it is evident that chitosan is effectively applicable in several fields, and chitosan would be a promising candidate in future applications as well. The discovery and progress of chitosan and its applications are exhibited in Scheme 3.

## 6. Key Challenges and Future Prospects

The production of chitosan involves the use of harsh chemicals, including strong acids and bases, and other methods, including enzymatic degradation, which is very expensive. The degree of deacetylation and the molecular weight of the obtained chitosan are highly variable. The dissolution of chitosan is not efficient and highly dependent on the molecular weight, solvent used, and degree of deacetylation, resulting in a poor yield. Standardization of the protocols for large-scale production is yet to be solved. Although chitosan has received a lot of interest for its use as a pesticide, herbicide, and fertilizer, further research is essential to fully comprehend this substance’s potential for the management of flora and fauna. Despite the research that has been done so far, it is still unclear how exactly chitosan works inside the plant cell. More transcriptome and proteome studies responsive to abiotic stress are thus needed to improve the use of chitosan in the regulation of plant growth. Although in vitro experiments for microbial or antioxidant activity or to assess the efficacy of delivery and encapsulation methods are useful, they must be supplemented with experimental applications in food preparation and under storage conditions. Even in simulated conditions, it is necessary to assess the bioactivity and bioavailability of drugs as well as their delivery across the gastrointestinal tract.

The use of chitosan as a packaging material could lessen global pollution despite several drawbacks in terms of thermal stability, barrier and mechanical characteristics, and production costs. Moreover, chitosan can be successfully combined with various organic and synthetic polymers, natural extracts, metal particles, and other substances to overcome these limitations. Chitosan is incompatible with hydrophobic drugs, and fewer toxicological responses have been reported. Though very limited challenges are available with the use of chitosan, in general it has been found to be promising in many applications. Clinical trials are essential to perform for larger populations rather than restricting the study to smaller numbered samples to reflect the practical usage of chitosan as a drug delivery system. The clinical trials should be more organized, using a proper time frame where proper control samples should be defined and used. Further studies on genomics, transcriptomics, and proteomics are required to understand the underlying mechanism of chitosan in plant, animal, and human cells. The majority of findings on gene therapy utilizing chitosan came from in vitro studies, and in vivo research is still required. Moreover, research is still needed to comprehend how the carriers’ properties affect cellular entrance and intracellular trafficking processes [335]. Most of the results on gene therapy using chitosan were obtained from experiments in vitro and further research is needed in vivo [336]. Membranes made of chitosan and albumin have been utilized for drug targeting, artificial skin, and hemodialysis. In these medicine-related fields, more advancements are expected in the near future.

Though functionalized and modified chitosan has been found to be effective in wastewater purification, more detailed studies, especially focusing on flow bed systems, are required. Furthermore, the applicability of chitosan to remove a broad spectrum of pollutants, including metal ions, dyes, pesticides, and pharmaceuticals, should be tested on artificial and real wastewater rather than testing only the most popular pollutants. Moreover, the efficiency of chitosan in removing many pollutants when they co-exist in water should be monitored. Chitosan could be effectively used in wound dressing and first aid applications, as well as in effective burn treatment. Furthermore, chitosan could be a promising alternative in regenerative medicine for applications such as bone repair, skin repair, and the regeneration of cartilage. Chitosan would be a favorable alternative for topical drug delivery for acute and chronic wounds, especially in patients who suffer from diabetes mellitus. Chitosan, along with common cosmetic valued products, such as algae extracts, fruit extracts, essential oils incorporated to gels, would be an attractive cosmetic product.

In addition to emerging fields such as nutraceuticals and cosmeceuticals, promising advancements were also occurring in the field of biomedicine [336]. In fact, chitosan and derivative-containing pharmaceutical formulations are also suggested for use in slimming products, body weight management, and cosmetics to increase the effectiveness of skincare products, among other uses. Clinical trials in different geographical areas are required to evaluate the effect of chitosan on these aspects. It is important to point out that, although numerous papers and patents have been reported in the last two decades, the applications of chitosan in the biomedical area are still limited, mainly due to the extreme difficulty to access sufficient purity and source reliability of the biopolymer. In addition, the development of new materials is rather limited, mainly due to their cost, which remains higher than that of petroleum-based polymers with similar properties. Finally, in vivo studies are currently limited. Further industrial developments are expected in the near future in the following domains: anticancer drugs, gene delivery, catalysis, sensor applications, wrapping materials and packaging, cosmetotextiles, and bio-imaging. The chitosan applications in the biomedical field are limited to laboratory-scale trials, despite the fact that many papers and patents have been reported over the past 20 years. This is primarily because it is extremely challenging to obtain the biopolymer with sufficient purity and source reliability. However, the development of novel materials is somewhat constrained, primarily because of their price, which is still higher than that of polymers made from petroleum that have comparable qualities [336]. Finally, there are currently few in vivo research and further studies are needed. Future industrial developments are expected in the following areas: anticancer medications, cosmetotextiles, bioimaging, sensor applications, gene delivery, catalysis, crosslinked materials, and nano materials, to fully utilise the potential of chitosan.

## 7. Conclusions

Due to its distinct properties, such as biocompatibility, biodegradability, low reactivity, and low allergenicity, chitosan has shown to be promising in multidisciplinary fields, including medicine, cosmetics, food, environmental remediation, and agriculture. However, the wide use of chitosan has been restricted to the lab scale experiments, owing to the low solubility, poor mechanical properties, inadequate rheological characteristics, low yield, and reported toxicity. Furthermore, elaborated research to overcome the above-mentioned challenges is essential to unveil the full potential of chitosan and its derivatives.

## Figures and Tables

**Table 1:** Commercially available chitosan related wound dressing products.

Commercial Producer	Product	Type	Material	References
Axiobio	Axiostat^®^	Sponge	100% chitosan	[94]
Trusetal	Chitoderm^®^ plus	Superabsorber	Strong superabsorber coated with chitosan	[95]
Sam Medical	ChitoSAM™ 100	Non-woven chitosan dressing spun directly from chitosan	100% chitosan	[96]
MedTrade	Celox™	Gauze (Celox Rapid, Celox Gauze) Granules (Celox A, Celox Granules)	Chito-R™ activated chitosan granules	[97]
Primex	ChitoClear^®^	Gel or liquid spray	ChitoClear^®^ positively charged chitosan (the purest chitosan possible)	[98]
Medline	Opticell^®^	Gelling fiber	Primarily composed of chitosan (Cytoform chitosan-based gelling technology)	[99]
Tricol Biomedical	ChitoFlex^®^ PRO	Hemostatic dressing active on both sides	chitosan based dressing	[100]
	ChitoGauze^®^ PRO	Chitosan-coated gauze		
	ChitoDot^®^	Double-sided hemostatic dressing		
	HemCon^®^ Bandage PRO	Hemostatic bandage		
	HemCon Patch^®^ PRO	Non-invasive hemostatic patch		
	HemCon^®^ Strip PRO	Hemostatic bandage		
BenQ Materials	ChitoClot Bandage	non-woven dressing	100% chitosan-based, non-woven with adhesive back sheet	[101]
	ChitoClot Pad	sponge	100% medical-grade chitosan	
	ChitoClot Gauze	Gauze		

**Table 2:** A summary of using chitosan for ophthalmology.

Property of Chitosan	Action	Application	References
High molecular weight Concentration of polysaccharides Non-irritant nature	To stop the lachrymal flow, thicken the solution and interact with the mucus’ negative charges. Increase ocular tolerability and precorneal residency time duration	Ocular carrier for topical administration of required medicament.	[133]
High tolerance	Longer contact time with ointments and ease of administration with accuracy and repeatability of the dose Ocular in situ gels are an effective substitute for traditional eye drops that have drawbacks such as rapid and thorough removal from the eye, which results in significant medication loss.	Ocular wound repair and increased permeability	[134]
Encapsulation effectiveness and particle size shape of nanoparticles Non-irritating	Release with controlled diffusion Drug-loaded nanoparticles keep their drug’s original crystallinity since the drug and polymer don’t interact.	Ocular delivery	[69]
Characteristics of mucoadhesion Crosslinking is intense in chitosan nanoparticles. Biocompatible and biodegradable	Enhances the drugs’ bioavailability and helps them penetrate the eye. Enhancements in the delivery site’s chitosan nanoparticle absorption, accumulation, and clearance	Creation of a formulation for a continuous medication releaseTreatment for glaucoma, age-related macular degeneration, viral and bacterial infections, and diabetic retinopathy	[132]
Exceptional mucoadhesive properties	While precorneal residency is prolonged, precorneal clearance could be hindered. Enhance distribution to the posterior eye segment by increasing the chance of adherence to corneal and conjunctival surfaces.	Age-related macular degeneration (AMD) and diabetic retinopathy (DR),Posterior eye drug delivery	[64]
Mucoadhesive, muco-penetration, and controlled release properties	Lower intraocular pressure in glaucomatous rabbits	Topical ophthalmic controlled drug deliveryTreatment of glaucoma	[66]
Charge-based nature and mucoadhesive qualities	Keep the anti-inflammatory diclofenac release under control while preserving or enhancing the lens’s physical characteristics	Increase the ocular bioavailability of diclofenac sodium already available	[65]
Low toxicity and good ocular tolerance Bioadhesion- and permeability-enhancing properties	Extending and improving medication retention on the surface of the eye. Serve as transmucosal drug transporters, allowing either the buildup of medicines in the corneal or conjunctival epithelia or the transportation of pharmaceuticals to the inner eye.	Ocular drug delivery	[135]
Penetration Enhancement	Enhance the drug’s concentration and its duration in the body to improve the accuracy of instilled drop solutions and dosing consistency.	Ocular Delivery Formulations, ocular inflammation	[134]

**Table 3:** Commercially available chitosan included cosmetic products.

Product/Commercial Name	Manufacturer	Application	References
Hydamer^TM^	Chitinor AS (Tromso, Norway)	Film forming and fixative agent, deodorizing	[149]
Triozan	Ovensa Inc. (Aurora, ON, Canada)	Penetration enhancer	[150]
Ritachitosan^®^	Rita Corporation (Crystal Lake, IL, USA)	Film-forming agent	[151]
Scalp-purifying micellar shampoo	Kristin Ess Hair (Los Angeles, CA, USA)	Shampoo	[152]
Curasan^TM^	Chemisches Laboratorium Dr. Kurt Richter GmbH (Berlin, Germany)	Film-forming agent	[153]
Brazillian Joia strengthening and smoothing shampoo	Sol de Janeiro, Inc. (New York, NY, USA)	Shampoo	[154]
Zenvivo^TM^	Clariant (Muttenz, Switzerland)	Film-forming agent, antimicrobial, deodorizing, moisturizer	[155]
Extra gentle conditioner	Kristin Ess Hair (Los Angeles, CA, USA)	Hair conditioner	[156]
KIOsmetine^®^	Kitozyme (Herstal, Belgium)	Film-forming agent, moisturizer	[157]
Herbal essences set me up gel	Procter and Gamble (Cincinnati, OH, USA)	Hair-styling gel	[158]
Chitosonic^®^ Acid	Personal Care Products Council (Washington DC, USA)	Antimicrobial, moisturizer	[159]
Re-vamp mid-length repair cream	Vernom Francois (Los Angeles, CA, USA)	Hair serum	[160]
ChitoClear^TM^	Primex Manufacturing Inc. (Langley, BC, Canada)	Film-forming agent	[161]
Anti-aging moisture lotion	Murad LLC (El Segundo, CA, USA)	Skin care	[162]
Everquat^TM^ Q50H	Sino Lion (Florham Park, NJ, USA)	Shining agent, antidandruff agent, hair growth promoter, anti-hair-loss agent	[163]
Ultimate miracle worker multi-rejuvenating cream	Philosophy (New York, NY, USA)	Skin care	[164]
Vinkocos p-6N	Vink Chemicals GmbH & Co. KG (Kakenstorf, Germany)	Film-forming and wetting agent, thickener, stabilizer	[165]
St. ives replenishing mineral therapy body lotion	Unilever (London, UK)	Skin moisturizer	[166]
Jeen-Chitosan	Jeen International (Fairfield, NJ, USA)	Film-forming agent, moisturizer	[167]

**Table 4:** The versatile films or coatings based on various formulas and uses.

Material Type	Additive	Property	References
Chitosan film	Cinnamon essential oils	Antimicrobial activity Mechanical strength Moisture barrier, Food shelf life extension	[246,247]
Chitosan film	Apricot kernel essential oils	Moisture barrier Antimicrobial activity Antioxidant activity Food shelf life extension	[248]
Chitosan film	Curcumin	Antimicrobial activity Antioxidant activity	[249]
Chitosan film	Purple-fleshed sweet potato extract	pH sensing Antioxidant activity	[250]
Chitosan film	Micro/nanoclay	Moisture barrier	[251]
Chitosan film	Cloisite 30B	Mechanical strength Moisture barrier	[252]
Chitosan film	Cloisite 15A	Thermal stability Oxygen barrier	[240]
Chitosan film	Citric acid	Cross-linking	[253]
Chitosan film	Glycerol	Plasticizing Flexibility	[248]
Chitosan film	Genipin	Mechanical strength Water resistance	[239]
Chitosan film	Amikacin	Antimicrobial activity	[254]
Chitosan coating	Chitosan nanoparticles	Antimicrobial activity	[255]
Chitosan coating	Silver-chitosan nanocomposite particles	Extended shelf life Antimicrobial activity	[245]
Chitosan coating	Zinc oxide	Moisture barrier Mechanical strength Antimicrobial activity	[256]
Chitosan film	Graphene oxide	Moisture barrier Mechanical strength Thermal stability	[257]
Chitosan film	Silver	Mechanical strength	[223]
Chitosan film	Multiwalled carbon nanotubes	Mechanical strength	[258]
Chitosan film	Sulfur	Antimicrobial activity Hydrophobicity Mechanical strength Moisture barrier	[259]
Chitosan film	Reduced graphene oxide	Mechanical strength Water resistance Antioxidant activity Electrical conductivity	[260]

**Table 5:** Effective outcomes against diverse abiotic challenges were found with chitosan-treated plants.

Plants	Stress	Method of Application	Effects	References
Common bean (*Phaseolus vulgaris* L.)	Drought	Foliar application on seedlings	Increased plant growth and yield	[296]
Grapevine (*V. vinifera*)	Drought	Dipping of stem cutting before planting	Maintained chlorophyll content	[301]
Basil (*Ocimum* *basilicum* L.)	Drought	Foliar application on seedlings	Enhanced plant growth	[302]
Coffee (*Coffea* *canephora var* *Robusta*)	Drought	Foliar application on seedlings	Increased mineral uptake, total chlorophyll content and increased the resistance to drought stress	[303]
Cowpea (*Vigna ungiculata*)	Drought	Foliar application on seedlings	Improved growth and yield	[295]
White clover (*Trifolium* *repens*)	Drought	Foliar application on seedlings	Enhanced the production of stress-responsive metabolites	[304]
*Thymus daenensis* Celak	Drought	Spraying before the flowering stage	Increased flowering and full bloom and reduced negative impact on dry matter and oil yield	[305]
Safflower (*Carthamus tinctorius* L.) *and sunflower* (*Helianthus annuus* L.)	Salt	Seed treatment	Stimulated seedling growth and and tolerance to oxidative stress, reduced enzyme activity and	[306]
*Plantago ovata*	Salt	Seed treatment	Increased roots hoot	[307]
*Plantago ovate* *Forsk*	Salinity	Seed treatment	increased roots hoot and alleviated salt stress	[307]
*O. sativa*	Salt	Seed treatment	Enhanced catalase and peroxidase enzyme production	[308]
*Vigna radiata*	Salt	Seed treatment	Stimulated morphological parameters	[309]
Maize (*Zea mays*)	Salt	Foliar application	Enhanced all growth critera	[310]
Wheat (*Triticum* *aestivum* L.)	Salt	Adding to the nutrient solution	Lessened adverse effect of salt stress	[311]
Wheat (*Triticum* *aestivum* L.)	Drought	Seed treatment	Improved chlorophyll content, growth parameter and antioxidant enzymes	[312]
Potato (*Solanum* *tuberosum*) var. Favourite	Drought	Foliar application	Alleviated drought stress damage in young potato	[313]
Apple (*Malus sieversii*)	Drought	Foliar application	Improved leaf membrane stability, increased antioxidant enzymes	[314]
Fenugreek (*Trigonella* *foenum-graecum* L.)	Salinity	Seed treatment	Improved leaf water content, photosynthetic parameters and lessened salt stress	[314]
